# Grid2 interacting protein is a potential biomarker related to immune infiltration in colorectal cancer

**DOI:** 10.1186/s40001-023-01468-x

**Published:** 2023-11-14

**Authors:** Jiajing Zhao, Jiazheng Quan, Weilin Chen, Xiaojun Xie

**Affiliations:** 1 Department of General Surgery, the First Affiliated Hospital of Shantou University Medical College, Shantou, 515000 China; 2 Marshall Laboratory of Biomedical Engineering, Institute of Biological Therapy, Shenzhen University Medical School, Shenzhen University, Shenzhen, 518055 China

**Keywords:** GRID2IP, Prognosis, Immune cell infiltration, Colorectal cancer, Drug sensitivity

## Abstract

**Background:**

Colorectal cancer (CRC) is one of the three deadliest malignant tumors in the world, posing a severe hazard to human health. Nonetheless, the 5-year survival rate for advanced CRC remains unsatisfactory. Grid2 interacting protein (GRID2IP) is a Purkinje fiber postsynaptic scaffold protein implicated in a number of signal transduction pathways in the nervous system. Previous studies have shown that Grid2 is closely related to the occurrence and prognosis of gastric cancer and many other diseases. Therefore, we aim to identify the relationship between GRID2IP and the occurrence and prognosis of CRC.

**Methods:**

Transcriptome data were retrieved from The Cancer Genome Atlas (TCGA) database to analyze the differential expression of GRID2IP in a variety of malignant tumors and then validate it by quantitative real time polymerase chain reaction(Q-PCR) and Western Blot in HT29 and SW480 cells. "DESeq2" package was used to analyze the differentially expressed genes (DEGs) between the high- and low-GRID2IP subgroups. In relation to DEGs, Gene Ontology (GO) enrichment and Kyoto Encyclopedia of Genes and Genomes (KEGG) analysis were performed. In addition, gene set enrichment analysis (GSEA) and single-sample gene set enrichment analysis (ssGSEA) were employed to examine DEGs-associated signaling pathways and GRID2IP-associated immune cell infiltration levels. Besides, overall survival (OS), disease-specific survival (DSS), and progression-free interval (PFI) were compared between the two subgroups using a Kaplan–Meier analysis. In addition, a prognostic model for GRID2IP and clinical characteristics was developed using the univariate Cox regression method. The "pRRophetic" package was applied to predict the drug sensitivity of different subgroups. Moreover, we also performed single-cell analysis of GRID2IP using the TISCH database.

**Results:**

GRID2IP is upregulated in CRC patients. The rise of GRID2IP inhibits the invasion of tumor-associated immune cells resulting in a lower immune score. In addition, high GRID2IP expression was associated with poor prognosis in different clinical subgroups. Analysis of single cells revealed that GRID2IP was predominantly expressed in immune cells, myofibroblasts, and cancerous cells. In terms of chemotherapy drug sensitivity, the subgroup with high GRID2IP expression was less sensitive to gemcitabine.

**Conclusions:**

Our results suggest that rising GRID2IP promotes tumor-associated immune cell infiltration and suggests adverse outcomes in CRC patients, which may be a useful biomarker for determining the prognosis of CRC and a potential target molecule for CRC therapy.

**Supplementary Information:**

The online version contains supplementary material available at 10.1186/s40001-023-01468-x.

## Introduction

Colorectal cancer, the third most prevalent malignant disease in the world, has had a substantial effect on people's health and increased the burden on patients' families. Annually, there are more than 1.85 million cases of CRC and 855,000 deaths [1, 2]. Numerous studies have elucidated the clinical features and molecular mechanisms of CRC, as well as the key signaling pathways associated with tumor growth and metabolism [3], the 3- or 5-year survival rate in Stage IV is far from expectation [4]. Metastasis has been confirmed as major cause of CRC fatality. Due to the liver's extensive portal and arterial blood supply, CRC metastasis occurs there the most frequently. [5]. According to the previous studies [6], approximately 25–30% CRC cases are clinically diagnosed as liver metastases and about 50% of CRC carriers will develop symptoms of liver metastases. Liver metastases may occur in some CRC patients following radical resection, resulting in more than 50% patient death [7]. With the increasing incidence of colon cancer [8], National Comprehensive Cancer Network guidelines proposal the use of surgery in combination with radiation and chemotherapy for CRC, and it is currently the standard of care agreed upon worldwide [9]. Though the treatment of CRC has improved, its prognosis is still unsatisfactory. Numerous previous studies, there are many biological markers related to the prognosis of CRC, such as *CXCL8*, *KLK8*, *IMPAT* [10–12]. Nevertheless, these biological markers and therapeutic targets have not significantly enhanced CRC patient survival. With the exploration of targeted drugs in recent years, immune checkpoint inhibitors (ICI) have achieved satisfactory safety and efficacy in the treatment of CRC patients. A variety of immunotherapy drugs, including pembrolizumab, nibulumab and ipilimumab, have been approved for the treatment of advanced CRC [13]. Early screening for CRC is very important to improve its prognosis [14]. As a consequence, the search for new biomarkers and their therapeutic targets is crucial to improve the prognosis of CRC.

Grid2 interacting protein (GRID2IP), a postsynaptic scaffold protein at synapses in parallel fibrous Purkinje cells, may link GRID2 to the actin cytoskeleton and various signaling molecules. According to previous reports, GRID2IP may be related to Alzheimer's disease [15], nervous system development [16] and hemophilia A [17]. Yao et al. indicated that GRID2 in whole blood samples of Parkinson's disease (PD) patients was significantly higher than that in healthy controls, and there was evidence that Grid2 was a biological indicator of PD [18]. In addition, GRID2IP can also increase the risk of irritable bowel syndrome [19]. Huang, et al. recently confirmed that GRID2 is closely associated to gut microbiota. Specifically, GRID2 deficiency in the mouse model resulted in changes in the species richness and composition of the gut microbiota, which affected the function of the gut microbiota and caused disorder of the neuroactive ligand–receptor interaction [20]**.** Therefore, we hypothesized that GRID2IP is associated with the occurrence and prognosis of intestinal tumors. Based on the TCGA database, we found that GRID2IP in many kinds of cancer patients were significantly different from that in normal controls. Specially, it was up-regulated in breast cancer and lung cancer but low expressed in head and neck squamous cell carcinoma (HNSC) and renal chromophobe cell carcinoma (KICH). In spite of this, few studies have reported the relationship between GRID2IP gene and CRC. The effect of GRID2IP on the prognosis of CRC and its effect on tumor microenvironment remains unclear.

In our study, we used transcriptome data retrieved from the TCGA database to identify the effect of GRID2IP on prognosis and tumor-associated immune cells in CRC. Functional enrichment analysis and protein interaction network were also utilized to explain the prognostic mechanism of GRID2IP. In addition, the K–M survival curves were used to visualize the relationship between GRID2IP and overall survival in CRC. Finally, a prognostic nomogram and its correction curve were established. Taken together, GRID2IP maybe a potential biomarker and drug target for predicting the prognosis of CRC, which is conducive to guiding clinical treatment strategy in CRCs.

## Materials and methods

### Data collection and preprocessing

Since most CRC are colorectal adenocarcinoma or colorectal adenocarcinoma, we downloaded their transcriptome data information from the TCGA database and combined them as colorectal adenocarcinoma (COADREAD). A total of 521 COADREAD cases with gene expression data (HTSeq-FPKM) and the detailed clinical information from COADREAD samples were downloaded from TCGA Repository, which includes 51 colorectal paracancerous tissues and 647 colorectal tissues. Incomplete or invalid data were excluded. Clinical features that were unavailable or unknown were considered missing values. In addition, transcripts per million reads (TMP) were derived from the level 3 HTSeq-FPKM data, which were used to the further study. Transcriptome data were divided into two subgroups based on the GRID2IP of each sample: GRID2IP-high and GRID2IP-low. Moreover, the normal colon tissues were extracted from GTEx database. Since all data for the study were obtained from freely accessible online databases, ethical approval and patient consent were not mandatory. The characteristics of all patients' clinical data are summarized in Additional file 2: Table S1. The complete research process diagram is summarized in Additional file 1: Fig. S2.

### The expression of GRID2IP between CRC and normal tissue

The differential expression of GRID2IP was calculated using disease status (tumor or normal) as a variable and presented in box plots and scatter plots. Receiver operating characteristic (ROC) curve analysis was utilized to assess GRID2IP's diagnostic value. The "pROC" and "ggplot2" packages were used to visualize the ROC curve.

### Quantitative real-time PCR

The total cell RNA of CCD18, HT29 and SW480 were extracted by Trizol reagent (Takara, Japan). Quantitative PCR results were performed by Hieff@ Qpcr SYBR®Green Master Mix (Yeasen, shanghai, China) and CFX96 Touch Real-Time PCR System (Bio-Rad, Hercules, CA, USA) on the basis of the manufacturer’s instructions. Gene-specific primer sequences: human GRID2IP: gene-specific primer sequences: human GRID2IP: (Forward primer 5’-CCATTTGCCAGTGACTCCGA-3’, Reverse primer 5’-TGTGCTGGAAGAAGCTCTCG-3’).

### Immunoblot analysis

CCD18, HT29 and SW480 cells were lysed by NP40 lysis buffer (1 × protease inhibitor mixture), centrifuged with 15000*g*, 12 min. Supernatants were added 1xloading buffer and then boiled 15 min. Mixtures were subjected to 6–10% SDS PAGE gel electrophoresis, transferring to PVDF membranes. The membrane was incubated with 5% skim milk for 1 h to block irrelevant proteins, following incubated with indicated antibody overnight at 4 °C. Next, the membrane was treated with secondary antibody for at room temperature 1.5 h. Proteins were visualized by Chemiluminescent reagents kits (Thermo Fisher Scientific, Waltham, MA, USA) and detected by FluorChem E (Cell Biosciences, USA), the antibody of GRID2IP purchased from abcam (Britain), GAPDH purchased from proteintech (China).

### Gene set enrichment analysis (GSEA)

GESA which is a computer algorithm based on gene expression matrix [21] can use a predefined genes concentrated to assess and the sorting of phenotypic correlation gene distribution trend, so as to determine its contribution to the phenotype. In our study, GSEA generated an ordered gene sequence of all genes according to their correlation with GRID2IP expression and then analyzed the significant survival differences between the GRID2IP-high and GRID2IP-low subgroups by GSEA (Each group was analyzed 1000 times). The expression profiles of our samples were input into GSEA as phenotypic markers, and pathways with GRID2IP enrichment in each phenotype were ranked using nominal *P* values and normalized enrichment score (NES). The “ClusterProfiler” package [22] was used to analyze the GSEA enrichment and visualization.

### DEGs analyze and enrichment of GRID2IP

According to the median GRID2IP expression levels (0–50% and 50–100%), a total of 647 COADREAD patients were divided into high GRID2IP and low GRID2IP subgroups. The R package "DESeq2" [23] was used to conduct Wilcoxon rank-sum test to identify DEGs groups between the high and low expressing GRID2IP, with log-fold change > 1.5 and adjusted *P* value 0.05 as the threshold. The R packages "Enhanced Volcano" and "PheatMap" were also applied to draw heatmap and volcano plot. Besides, R package ggplot2 was used to visualize the enrichment analysis of differential genes in paracancerous and tumor tissues analyzed by “ClusterProfiler” package. The “GOplot” package is applied to calculate the zscore [24].

### Functional enrichment analysis of Immune microenvironment

Single sample GSEA from R package “GSVA” [25] was powered to evaluate the immune infiltration level. The relative tumor invasion level of immune cells was quantitatively assessed by integrating the type signature gene list of published gene expression levels [26]. The 24 types of immune cells were performed to evaluate the immune cells enrichment in tumor tissues. To detect immune cell infiltration and expression levels of different GRID2IP mRNA, Wilcoxon rank sum test and Pearson correlation analysis were performed. TISCH online database(http://tisch.comp-genomics.org/) is applied to analyze the relationship between GRID2IP and TME. In the aspect of designing database parameter, we focus on the Maj-Lineage (Cell type). CRC (Cancer type); No parameter set (Cell type); All (Lineage for calculating correlation); No treatment(Treatment); Primary(Primary/Metastatic). In addition, the stromal score, ESTIMATE score and immune score was calculated using "estimateScore" algorithm. Furthermore, lollipop plot was generated using "ggplot2" package to delineate the correlation between GRID2IP and immune checkpoint inhibitor-related genes.

### Clinical features analysis and prognosis assessment

All statistical analyses of this study were performed in the R package (v.4.2.1). Wilcoxon rank sum test and logistic regression were applied to clarify the relationship between clinical features and GRID2IP. Cox regression and Kaplan–Meier methods were used to investigate the clinical features associated with OS, DSS and PFI in TCGA database patients. A multivariate Cox analysis was employed to examine the influence of GRID2IP expression level on survival and other clinical features (stage, subtype, status of distant metastases, and histological grade). The median determined the cutoff value for the GRID2IP expression. The Kaplan–Meier analysis and the two-sided log-rank test were used to calculate the differences in 10-year OS, PFI, and DSS between the high and low GRID2IP subgroups. The nomogram based on the Cox regression model was created using the independent prognostic indicators discovered by multivariate analysis to estimate the odds of survival for 1, 3, 5 years, respectively. The "rms" package was employed to create nomograms with calibration plots and clinical features. Calibration plots were performed to analyze the nomogram prediction probabilities against observed events. Of note, the 45° line represented the actual value. C index represents the accuracy of nomogram prediction and the accuracy of different prognostic factors. All statistical tests in this investigation were two-tailed.

### Statistical analysis

The data of quantitative PCR were presented with mean ± SD with at least three independent experiments. GraphPad Prism (v.8.0) was used to conduct a student's *t* test between groups, and *p* < 0.05 (two-sided) was considered statistically significant. The Wilcoxon test was applied to examine the differences between the two groups' expression. Chi-square test was used to investigate the correlation between GRID2IP expression and clinical features between the two subgroups.

## Results

### GRID2IP was abnormally elevated in CRC

**Fig. 1:**
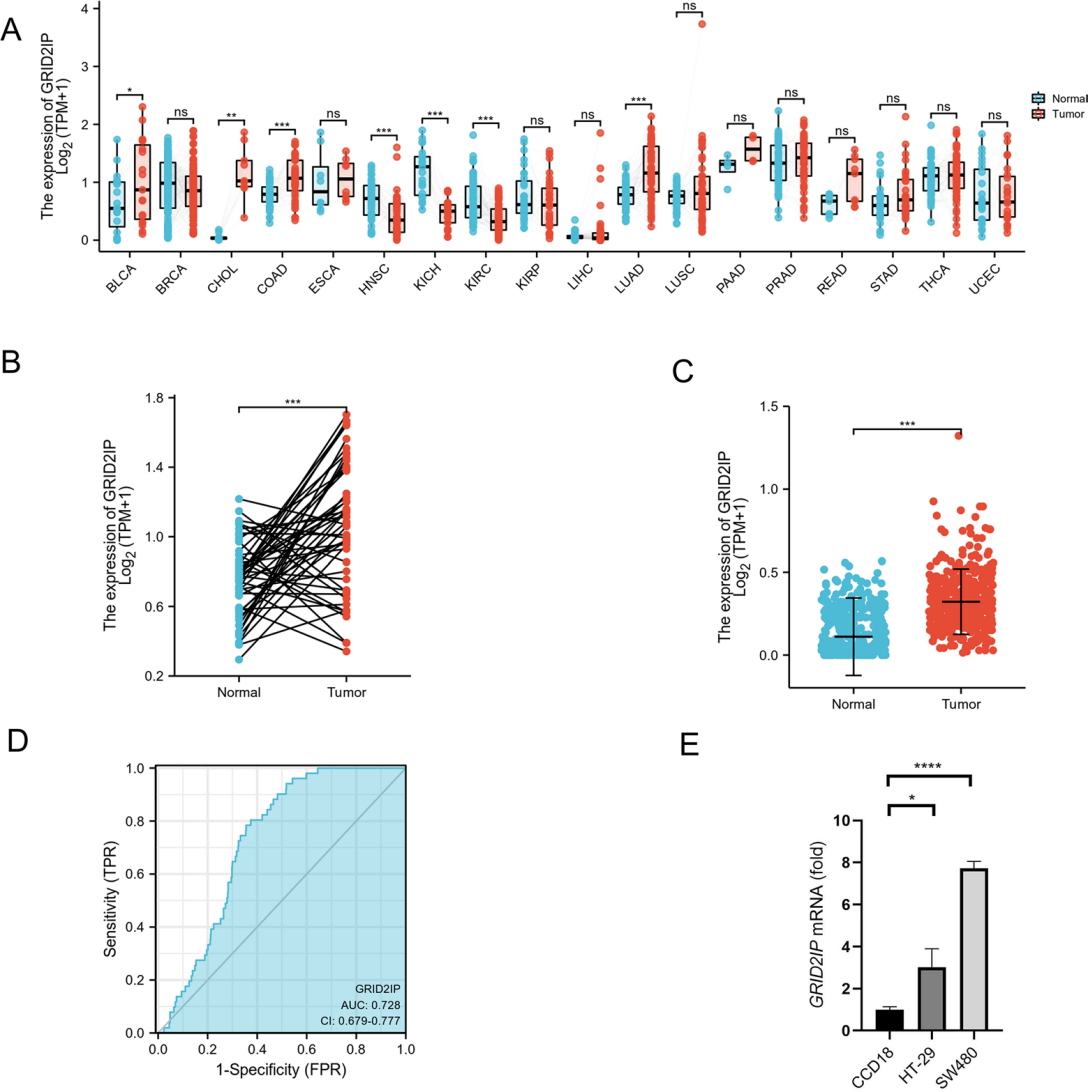
GRID2IP is up-regulated in CRC.** A** Differential expression levels of GRID2IP in normal tissue samples from healthy people in GETx database and tumor tissue samples in TCGA in pan-cancer. **B** Differential expression levels of GRID2IP in paired paracancerous normal tissues (*n* = 51) and tumor tissues (*n* = 383) derived from COADREAD in TCGA. **C** Expression of GRID2IP in normal tissues(*n* = 359) from GETx and tumor tissues (n = 383) from COADREAD patients in TCGA. **D** ROC curve used to identify COADREAD tissue with the value of GRID2IP. **E** mRNA level of GRID2IP in CCD18, HT-229 and SW480 cells. Data are showed as mean ± SD. *p < 0.05, **p < 0.01, ***p < 0.001

Aim to investigate the general expression of GRID2IP in various cancers, we first analyzed pan-cancer, compared the expression of GRID2IP in GTEx combined with TCGA samples, and performed Wilcoxon rank sum test analysis on matched samples in TCGA. The results show that GRID2IP is found in bladder carcinoma(BLCA), cholangiocarcinoma (CHOL), colon adenocarcinoma(COAD), lung adenocarcinoma(LUAD) was highly expressed and low expressed in KICH, KIRC and HNSC (Fig. 1A). Second, we analyzed the expression of GRID2IP in adjacent tissues and COADREAD samples in TCGA cohort. In the paired sample, we discovered that GRID2IP expression was substantially higher in tumor tissues than in normal tissues. (Fig. 1B). In the unpaired sample, we get similar results (Fig. 1C). Moreover, ROC curve was applied to identify the differential performance of GRID2IP, and the result showed that the area under the curve(AUC) value was 0.728, indicating that GRID2IP gene has a potential role in differentiating normal tissues from tumor tissues (Fig. 1D).

To further ensuring the GRID2IP was increasing in CRC, we detected the protein level and mRNA level of GRID2IP in the CCD18, HT-29 and SW480 cells. We found that the mRNA level of GRID2IP in the HT-29 and SW480 cells was higher than that in CCD18 cells (Fig. 1E). Moreover, the GRID2IP protein was raising in the HT-29 and SW480 cells, comparing with CCD18 cells (Additional file 1: Fig. S1A, B). It’s suggesting that the GRID2IP’s expression was higher in the colorectal cancer line cells.

### GRID2IP related gene enrichment analysis and DEGs screening

**Fig. 2:**
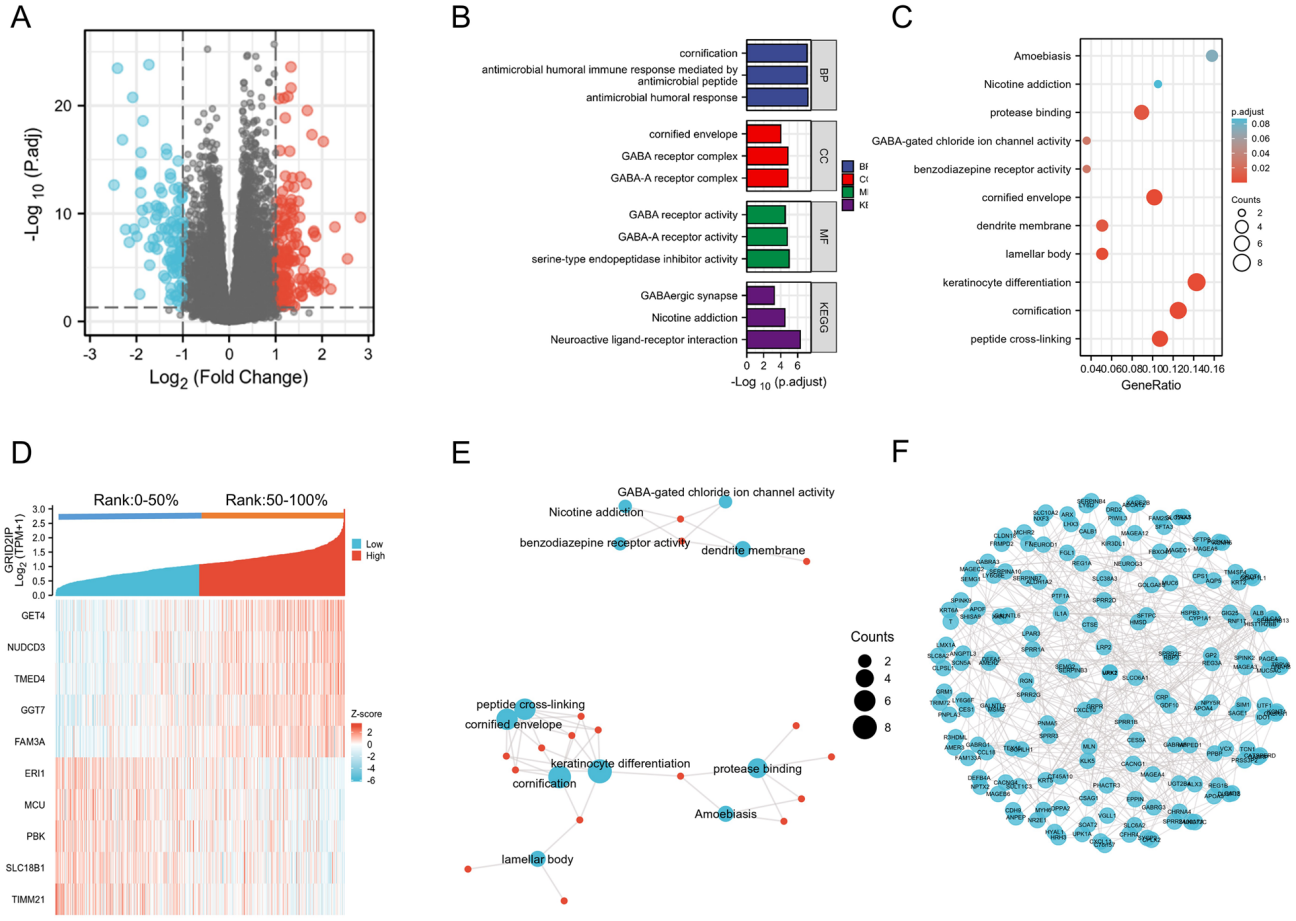
Differential gene expression and enrichment analysis between high and low GRID2IP expression groups. **A** Volcano map of DEGs between high and low GRID2IP expression groups. **B, C** Bar and Bubble Plots for KEGG and GO Analysis of DEGs. **D** Heatmap of the 10 DEGs most associated with GRID2IP. **E**, **F** Network diagram of DEGs interacting proteins

“DESeq2” package was applied to perform gene differentiation analysis on 322 samples with high GRID2IP expression and 322 samples with GRID2IP-low. Altogether, 254 differentially expressed genes were found, of which 156 were upregulated and 98 down-regulated (Fig. 2A). Statistically, the difference between the two categories was significant (*p* < 0.05, |log2 FC|> 1.0). “ggplot2” package was employed to further analyzed the DEGs in the HTseq-counts format, and the relevant expression levels of the top 10 differentially expressed genes are given in Fig. 2D. 50 items of GRID2IP-related DEGs are shown in Additional file 3: Table S2.

### GO&KEGG analysis

To investigate the functional enrichment information of GRID2IP interacting genes in greater depth, “ClusterProfiler” package was applied to enrich GO and KEGG. The results showed that GRID2IP-related DEGs are involved in many biological processes (BP), such as antimicrobial humoral response, cornification, keratinocyte differentiation, keratinization, epidermal cell differentiation. Besides, it also involved in cellular component(CC) and molecular function (MF). CC includes cornified envelope, lamellar body, dendrite membrane, neuron projection membrane, GABA-A receptor complex. MF includes protease binding, GABA-gated chloride ion channel activity, GABA receptor activity, inhibitory extracellular ligand-gated ion channel activity, structural constituent of epidermis. In addition, KEGG analysis of GRID2IP-related genes was related to nicotine addiction, GABAergic synapse and neuroactive ligand–receptor interaction (Fig. 2B, C). The results are network visualized in Fig. 2E. Detailed results of the GO and KEGG analyses are presented in Additional file 4: Table S3.

### Construction of PPI protein interaction network

To further investigate the interaction relationship between DEGs genes, 254 DEGs (154 genes were upregulated and 98 were downregulated.) were used to construct PPI interaction network by STRING database (The top 50 associated interacting proteins are presented in Additional file 6: Table S5), and the relationship threshold was set at 0.9. A total of 325 interacting protein pairs were selected to be shown in network spheremap (Fig. 2F).

### Identification of GRID2IP-related signaling pathways

**Fig. 3:**
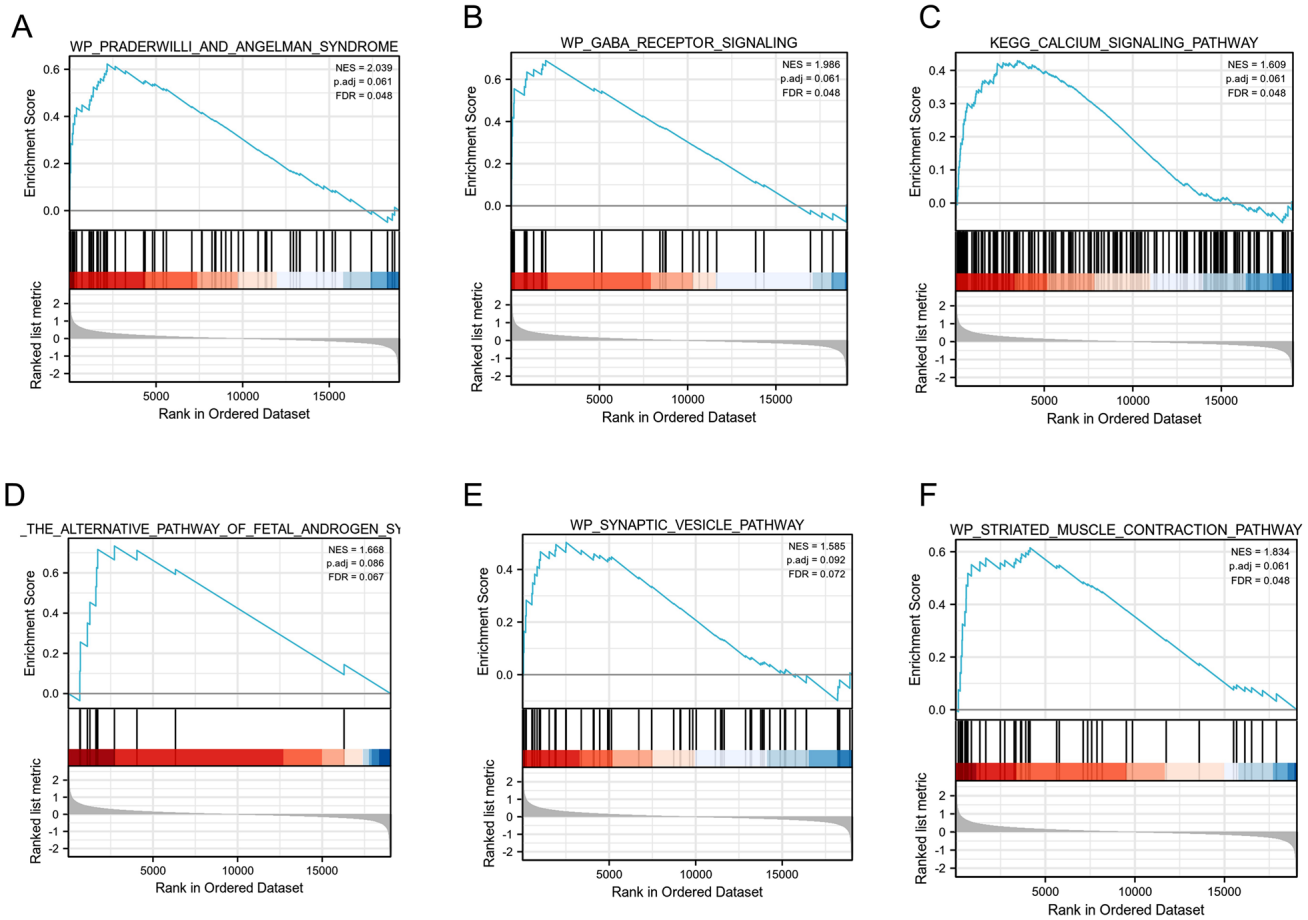
GSEA analysis. Differential enrichment of biological processes and signaling pathways, among which the stronger correlations include angelman syndrome (**A**), GABA signaling pathway (**B**), calcium signaling pathway (**C**), the alternative signaling pathway of fetal androgen synthesis (**D**), vesicle pathway (**E**), striated muscle contraction signaling pathway (**F**)

To identify GRID2IP-related signaling pathways in COADREAD, we performed GSEA analysis between data sets with GRID2IP-high and GRID2IP-low to identify statistically significant differences (adjusted *p* < 0.05, FDR < 0.25, MSigDB Collection (C2. cp. v7.2. symbols. GMT [Curated]). According to its normalized enrichment score (NES), the signal pathway with the highest degree of enrichment was chosen. The core molecules in the gene concentration were mainly concentrated in GRID2IP-high group. In addition, the differentially enriched pathways in GRID2IP-high group were calcium, muscle contraction, and GABA signaling pathway, the alternative signaling pathway of fetal androgen synthesis and vesicle pathway. (Fig. 3A–F). The results of the GSEA analysis for the top 50 items (ranked from largest to smallest based on NES) are presented in Additional file 5: Table S4.

### Association of GRID2IP and immune cell infiltration in CRC

**Fig. 4:**
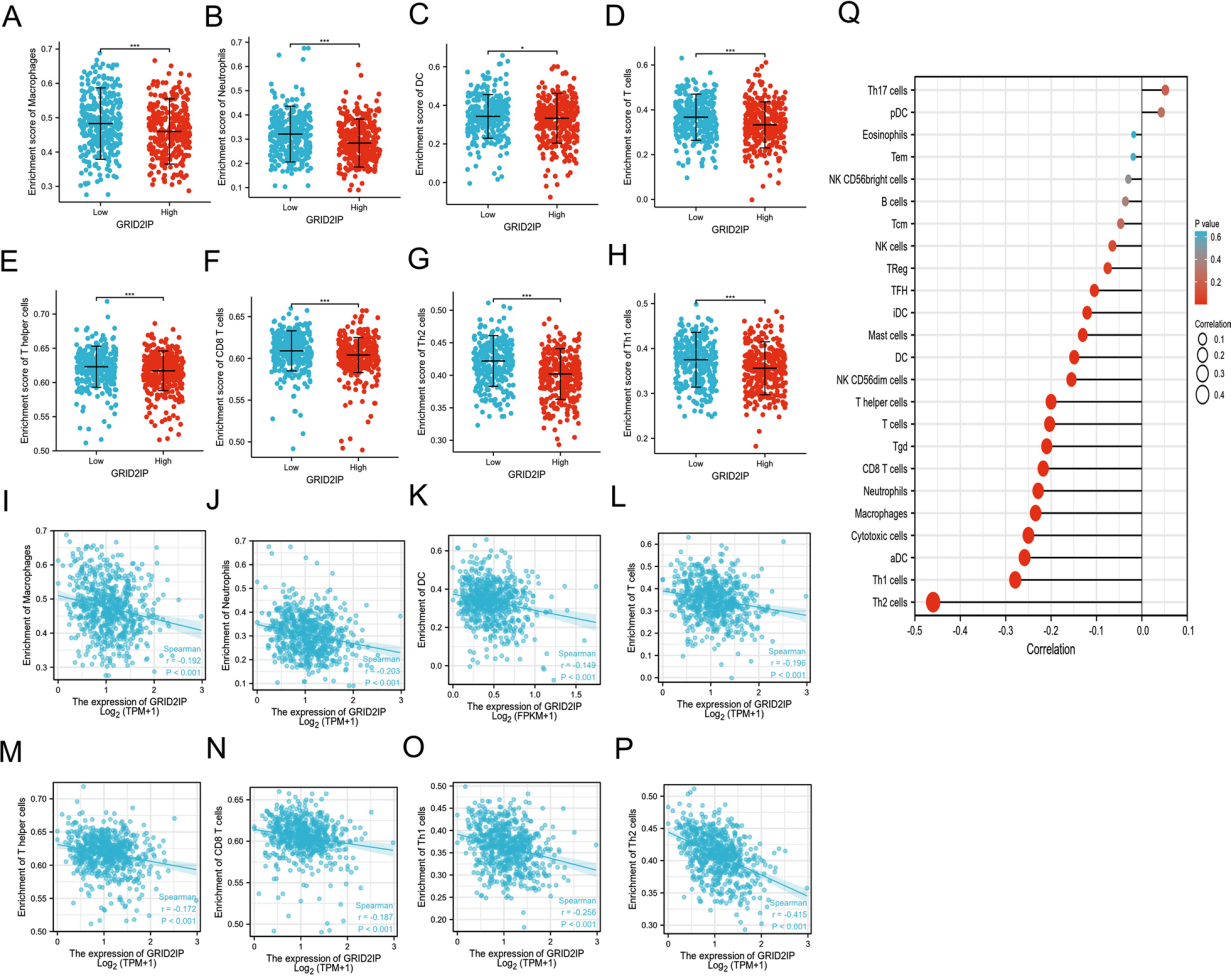
GRID2IP-related immune cell infiltration in CRC. **A–H** Infiltration levels of different immune cells in high and low expression GRID2IP groups. Tumor-associated immune cells with statistically significant differences were Macrophages (**A**), Neutrophils (**B**), DC cell (**C**), T cells (**D**), T helper cells (**E**), CD8 + T cells (**F**), Th2 cells (**G**), Th1 cells (**H**). **I**–**P** Scatter plots of corresponding immune cells. **Q** Correlation between relative abundance of 24 immune cells and GRID2IP expression. The size of the circle dots in the figure represents the absolute Spearman's correlation coefficient value. Data are showed as mean ± SD. *p < 0.05, **p < 0.01, ***p < 0.001

Considering the above GO and KEGG enrichment analysis found that GRID2IP may be involved in tumor immune regulation, ssGSEA was applied to further analyzed the relationship between 24 kinds of immune cell infiltration and GRID2IP by the spearman correlation. The results showed that the enrichment level of immune cells in GRID2IP-high subgroup was lower than that in GRID2IP-low subgroup. Moreover, the involvement of high-expression GRID2IP in tumor immunity was mainly related to T cells and innate immune cells, including T cells, CD8 + T cells, Macrophages, Neutrophils, DC cell, T helper cells, Th1 cells, Th2 cells. However, there was no significant effect on the enrichment level of B cells (Fig. 4A–H). Similarly, the scatter plot results showed that T cells, CD8 + T cells, Macrophages, Neutrophils, T helper cells, Th1 cells, Th2 cells DC cells are negatively correlated with GRID2IP (Fig. 4I–P). A GRID2IP-related lollipop chart of 24 immune cells was made to reflect the correlation between each type of immune cell and GRID2IP (Fig. 4Q). In addition, ESTIMATE score and immune score were lower in the GRID2IP-high subgroup, while stromal score found no significant difference (Additional file 1: Fig. S3A). Of note, GRID2IP was found to be negatively correlated with most immune checkpoint inhibitor-related genes (including *THFSF9, TNFRSF9, HAVCR2, CD80, CD86, NRP1 LAIR1, CD200 and CD276*) with *BTNL2, TNFSF15* were positively correlated (Additional file 1: Fig. S3B). Similarly, GRID2IP was negatively associated with most HLA-related genes, including *HLA-A, HLA-C, HLA-DOA, HLA-DPB1, HLA-DQA1, HLA-DRB5, DQB1, HLA-DPA1, HLA-DMA and HLA-DMB* (Additional file 1: Fig. S3C).

**Fig. 5:**
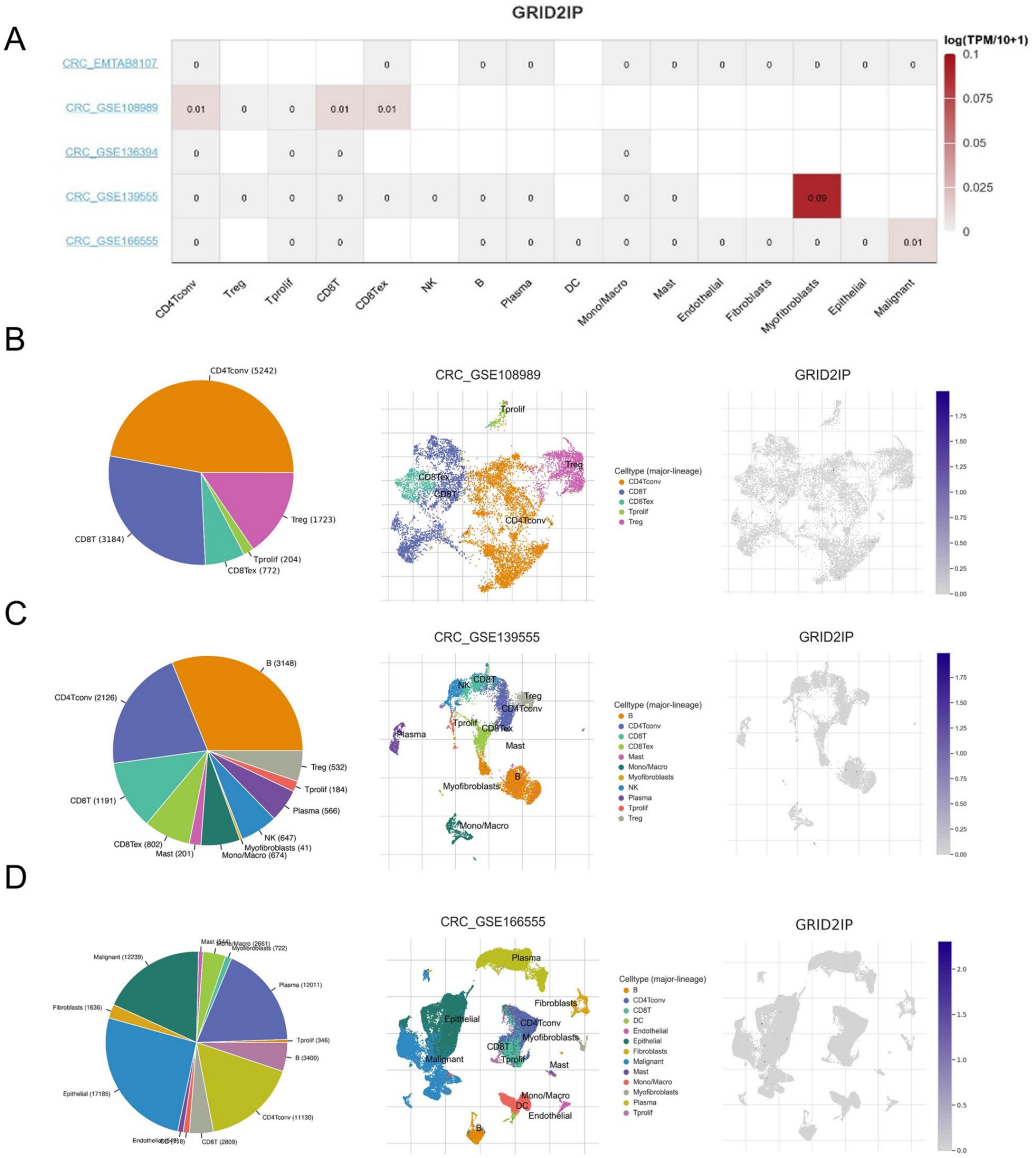
Correlation between GRID2IP and immune microenvironment at the single-cell level. **A** GRID2IP expression heatmap of single cell analysis in different data sets from the TISCH database. **B–D** GRID2IP expression in different cell types based on GSE108989, GSE139555 and GSE166555

Single-cell analysis was used to understand the correlation between GRID2IP and TME. We found three GRID2IP-related single-cell analysis results in the TISCH database. The results suggest that GRID2IP is mainly expressed in immune cells, myofibroblast and malignant cells (Fig. 5A). Specifically, in CRC-GSE108989, GRID2IP was mainly expressed in CD4 + T cells and CD8 + T cells (Fig. 5B). In CRC-GSE139555, GRID2IP was mostly expressed in myofibroblasts (Fig. 5C). In CRC-GSE166555, GRID2IP was mostly expressed in malignant cells (Fig. 5D).

### GRID2IP expression level is correlated with clinical features

**Fig. 6:**
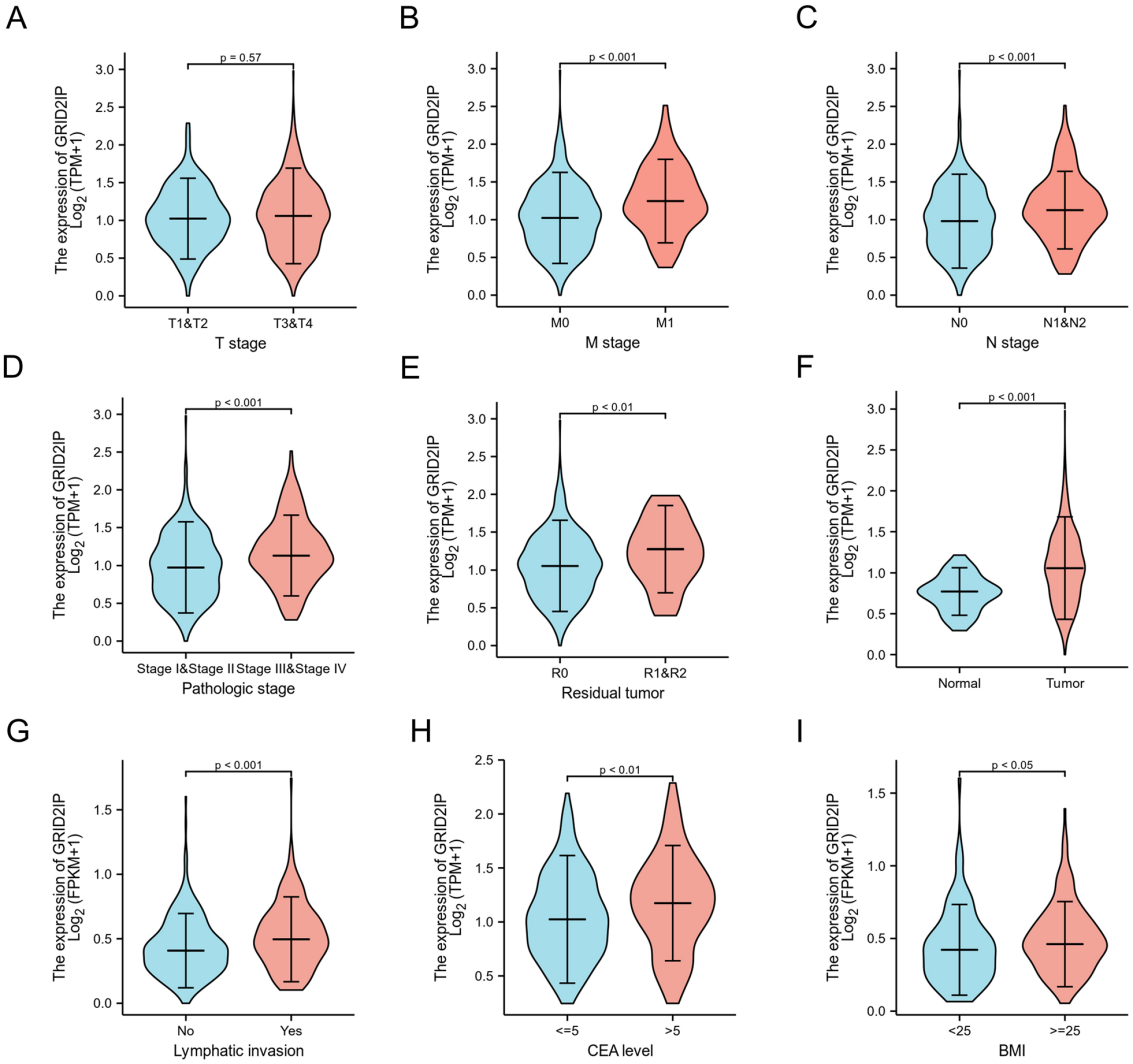
GRID2IP is associated with clinical characteristics. **A** No statistically significant difference in T stage. **B**–**I** Clinical characteristics with statistically significant difference are M-stage **(B)**, N-stage **(C)**, pathologic stage (**D**), residual tumor (**E**), status (**F**), lymphatic invasion (**G**), CEA level (**H**), BMI (**I**)

To further elucidate the relationship between GRID2IP and COADREAD patient clinical characteristics, we performed statistical analysis on 644 COADREAD patients with complete clinical data using the logistic regression method. The cohort consisted of 301 female and 343 male patients, with an average age of 68 (IQR: 57, 75.75) in the high GRID2IP group and 68 (IQR: 60, 76) in the low GRID2IP group. The results showed that up-regulation of GRID2IP was related to worse clinical features, which includes T-stage, N-stage, M-stage, pathology stage, BMI, residual tumor, lymphatic invasion, CEA level, tumor status (Fig. 6A–I). The complete logistic regression analysis results of GRID2IP are summarized in Table 1. In addition, the assessment of risk factors for differences in GRID2IP expression levels among difgroups is shown in Table 2. Based on the results of a logistic regression analysis, we investigated the influence of various risk factors on the CRC prognosis. Univariate Cox regression revealed an association between elevated GRID2IP expression and unfavorable OS [hazard ratio (HR): 1.486;95% confidence interval (CI) 1.047–2.109; *P* = 0.027], DSS (HR: 1.991; Cl 1.248–3.175; *P* = 0.004) and PFI (HR: 1.398; Cl 1.028–1.902; *P* = 0.033) (Table 3 and Additional file 7: Table S6 and Additional file 8: Table S7). To investigate the influence of related factors on survival, we performed Multivariate Cox regression analysis of pathological stage(TNM), grade, BMI, CEA level as well as residual tumor status. The results are shown in (Table 3 and Additional file 7: Table S6 and Additional file 8: Table S7). Notably, the P value results may be affected by the insufficient sample size included in Multivariate Cox regression analysis.

**Table 1:** GRID2IP expression association with clinical characteristics (logistic regression)

Characteristics	Total (*N*)	Odds Ratio(OR)	*P* value
T stage (T3&T4 vs. T1&T2)	641	1.231 (0.838–1.812)	0.291
N stage (N1&N2 vs. N0)	640	1.789 (1.305–2.460)	< 0.001
M stage (M1 vs. M0)	564	2.421 (1.508–3.970)	< 0.001
Pathologic stage (Stage III&Stage IV vs. Stage I&Stage II)	623	2.001 (1.453–2.765)	< 0.001
Age (> 65 vs. ≤65)	644	1.079 (0.790–1.475)	0.633
Residual tumor (R1&R2 vs. R0)	510	1.815 (0.954–3.581)	0.075
CEA level (> 5 vs. ≤5)	415	2.025 (1.350–3.059)	< 0.001
Primary therapy outcome (PD&SD&PR vs. CR)	312	1.160 (0.645–2.100)	0.621
Lymphatic invasion (Yes vs. No)	582	2.055 (1.468–2.889)	< 0.001
BMI (≥ 25 vs. < 25)	329	1.645 (1.035–2.627)	0.036

**Table 2:** Assessment of risk factors for differences in GRID2IP expression levels among different subgroups

Characteristic	Levels	Low expression of GRID2IP	High expression of GRID2IP	p
*n*		322	322	
T stage, *n* (%)	T1	10 (1.6%)	10 (1.6%)	0.132
	T2	61 (9.5%)	50 (7.8%)	
	T3	222 (34.6%)	214 (33.4%)	
	T4	28 (4.4%)	46 (7.2%)	
N stage, *n* (%)	N0	206 (32.2%)	162 (25.3%)	< 0.001
	N1	75 (11.7%)	78 (12.2%)	
	N2	38 (5.9%)	81 (12.7%)	
M stage, *n* (%)	M0	250 (44.3%)	225 (39.9%)	< 0.001
	M1	28 (5%)	61 (10.8%)	
Pathologic stage, *n* (%)	Stage I	58 (9.3%)	53 (8.5%)	< 0.001
	Stage II	142 (22.8%)	96 (15.4%)	
	Stage III	83 (13.3%)	101 (16.2%)	
	Stage IV	27 (4.3%)	63 (10.1%)	
Primary therapy outcome, *n* (%)	PD	18 (5.8%)	15 (4.8%)	0.457
	SD	2 (0.6%)	3 (1%)	
	PR	5 (1.6%)	11 (3.5%)	
	CR	129 (41.3%)	129 (41.3%)	
Gender, *n* (%)	Female	149 (23.1%)	152 (23.6%)	0.874
	Male	173 (26.9%)	170 (26.4%)	
Race, *n* (%)	Asian	6 (1.5%)	6 (1.5%)	0.030
	Black or African American	24 (6.1%)	45 (11.4%)	
	White	164 (41.6%)	149 (37.8%)	
Age, *n* (%)	≤65	141 (21.9%)	135 (21%)	0.691
	> 65	181 (28.1%)	187 (29%)	
Weight, *n* (%)	≤ 90	122 (35.1%)	122 (35.1%)	0.091
	> 90	41 (11.8%)	63 (18.1%)	
BMI, *n* (%)	< 25	60 (18.2%)	47 (14.3%)	0.047
	≥ 25	97 (29.5%)	125 (38%)	
Height, *n* (%)	< 170	76 (23.1%)	83 (25.2%)	1.000
	≥170	81 (24.6%)	89 (27.1%)	
Neoplasm type, *n* (%)	Colon adenocarcinoma	240 (37.3%)	238 (37%)	0.928
	Rectum adenocarcinoma	82 (12.7%)	84 (13%)	
Colon polyps present, *n* (%)	No	105 (32.5%)	119 (36.8%)	0.630
	Yes	50 (15.5%)	49 (15.2%)	
History of colon polyps, *n* (%)	No	190 (34.2%)	187 (33.7%)	1.000
	Yes	90 (16.2%)	88 (15.9%)	
Lymphatic invasion, *n* (%)	No	198 (34%)	152 (26.1%)	< 0.001
	Yes	90 (15.5%)	142 (24.4%)	
Residual tumor, *n* (%)	R0	235 (46.1%)	233 (45.7%)	0.136
	R1	3 (0.6%)	3 (0.6%)	
	R2	12 (2.4%)	24 (4.7%)	
CEA level, *n* (%)	≤ 5	140 (33.7%)	121 (29.2%)	< 0.001
	> 5	56 (13.5%)	98 (23.6%)	
Perineural invasion, *n* (%)	No	89 (37.9%)	86 (36.6%)	0.193
	Yes	24 (10.2%)	36 (15.3%)	
OS event, *n* (%)	Alive	268 (41.6%)	247 (38.4%)	0.049
	Dead	54 (8.4%)	75 (11.6%)	
DSS event, *n* (%)	Alive	284 (45.7%)	260 (41.8%)	0.005
	Dead	27 (4.3%)	51 (8.2%)	
PFI event, *n* (%)	Alive	250 (38.8%)	229 (35.6%)	0.071
	Dead	72 (11.2%)	93 (14.4%)	
Age, median (IQR)		68 (60, 76)	68 (57, 75.75)	0.445
Height, median (IQR)		170 (160.02, 175.2)	170 (162.38, 179)	0.421
Weight, median (IQR)		76 (64.1, 90.35)	83 (67.2, 94.7)	0.032
BMI, median (IQR)		26.67 (23.14, 30.42)	27.4 (24.82, 33.15)	0.038

**Table 3:** Univariate and multivariate Cox regression analyses of prognostic covariates (overall survival) in patients with CRC

Characteristics	Total (*N*)	Univariate analysis		Multivariate analysis	
		Hazard ratio (95% CI)	*P* value	Hazard ratio (95% CI)	*P* value
N stage	639				
N0	367	Reference			
N1	153	1.774 (1.131–2.781)	0.013	0.021 (0.001–0.507)	0.017
N2	119	3.873 (2.588–5.796)	< 0.001	0.024 (0.001–0.592)	0.022
M stage	563				
M0	474	Reference			
M1	89	3.989 (2.684–5.929)	< 0.001	2.083 (0.516–8.404)	0.302
Pathologic stage	622				
Stage I & Stage II	348	Reference			
Stage III & Stage IV	274	2.988 (2.042–4.372)	< 0.001	83.444 (2.290–3040.287)	0.016
Age	643				
≤65	276	Reference			
>65	367	1.939 (1.320–2.849)	< 0.001	3.738 (0.794–17.602)	0.095
BMI	329				
<25	107	Reference			
≥25	222	0.649 (0.394–1.069)	0.090	0.715 (0.200–2.555)	0.605
CEA level	414				
≤5	260	Reference			
>5	154	2.620 (1.611–4.261)	< 0.001	0.865 (0.206–3.627)	0.842
Residual tumor	509				
R0	467	Reference			
R1 & R2	42	4.609 (2.804–7.577)	< 0.001	68.163 (6.142–756.470)	< 0.001
Lymphatic invasion	581				
No	349	Reference			
Yes	232	2.144 (1.476–3.114)	< 0.001	3.049 (0.686–13.557)	0.143
GRID2IP	643				
Low	321	Reference			
High	322	1.486 (1.047–2.109)	0.027	1.147 (0.305–4.316)	0.839

The Hazard ratio of the Reference group is the Reference and the other groups is compared with the reference group to obtain the HR value

### Upregulated GRID2IP predicts poor clinical prognosis in CRC

**Fig. 7:**
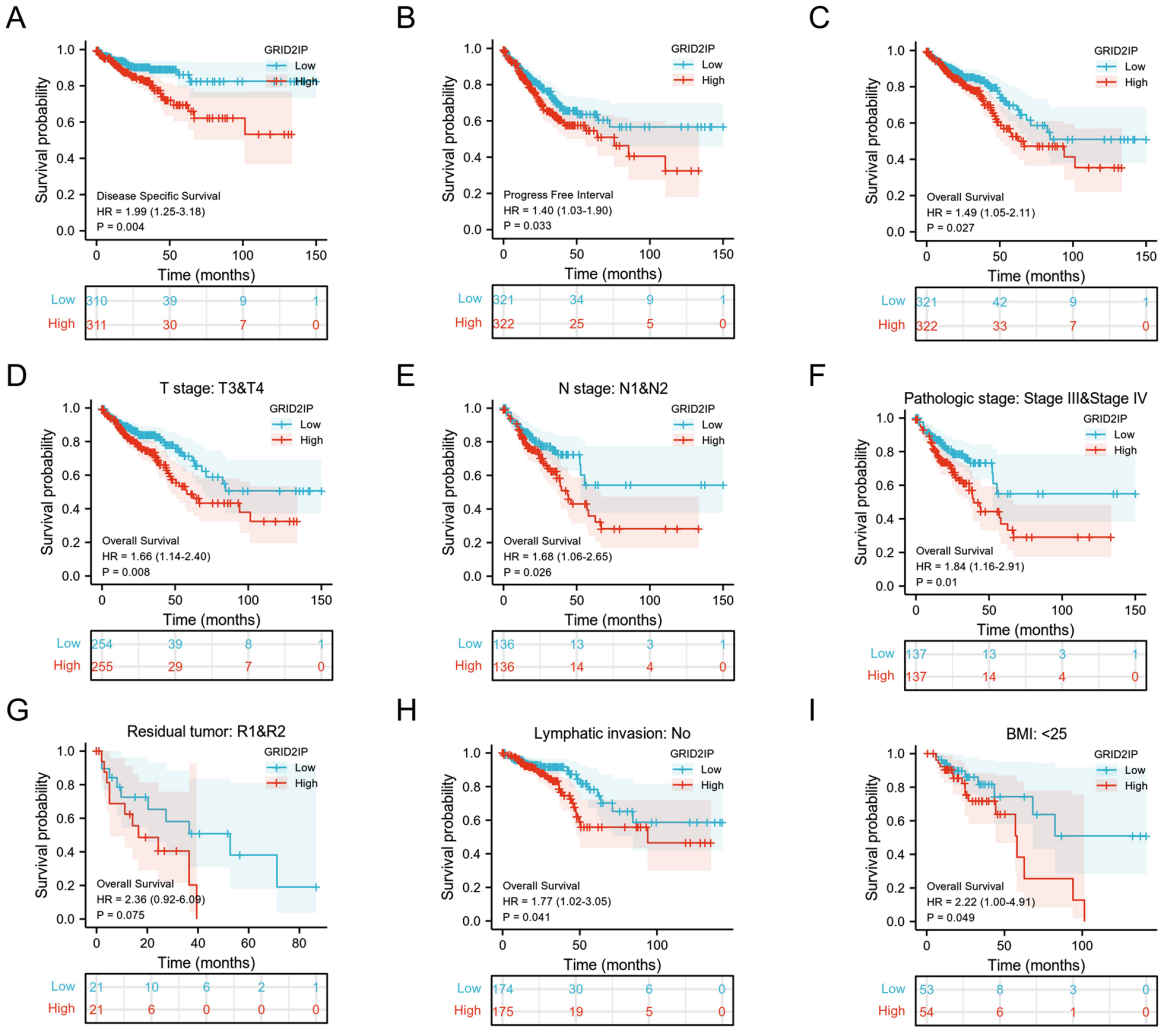
Kaplan–Meier survival curves comparing high and low expression of GRID2IP in CRC. **A**–**C** Overall Survival (OS), Disease Specific Survival (DSS), Progress Free Interval (DFI) of CRC patients with high and low expression of GRID2IP. **D**–**I** KM survival curves (OS) of high and low GRID2IP group in different clinical subgroups. There are statistically significant differences, including T-stage (T3&T4) (**D**), N-stage (N1&N2) (**E**), Pathologic stage (Stage III & Stage IV) (**F**), residual tumor (R1&R2) (**G**), lymphatic invasion (No) (**H**) and BMI (< 25) (**I**)

To clarify the relationship between GRID2IP e and survival prognosis of CRC patients, we used Kaplan–Meier plotter analysis to observe the 1-, 3-, and 5-year survival rates between the GRID2IP-high and GRID2IP-low subgroups. The KM curves showed that the 1-, 3-, and 5-year OS, PFI and DSS of the high-GRID2IP subgroup were lower than those of the low-expression GRID2IP group (Fig. 7A–C. Furthermore, to find theeen prognosis and GRID2IP expression level between each subgroup, we performed KM curve analysis within different subgroups. The results showed that in T3&T4 stage subgroup (HR: 1.66; CI 1.14–2.40; *P* = 0.008), N1&N2 stage (HR: 1.68; CI 1.06–2.65; *P* = 0.026), Pathology Stage III & Stage IV (HR: 1.84; CI 1.16–2.91; *P* = 0.01) and Residual (R1&R2) tumor (HR: 2.36; CI 0.92–6.09; *P* = 0.075), the OS of the GRID2IP-high group were lower than those of GRID2IP-low subgroup (Fig. 7D–G). The Overall Survival prognostic value of GRID2IP in various CRC subgroups are shown in Table 4. This indicates that GRID2IP plays a favorable role in predicting prognosis in patients with poor clinicopathological grades. Notably, the OS of the GRID2IP-high subgroup without lymph node infiltration and BMI < 25 was also significantly decreased compared with the GRID2IP-low group (Fig. 7H–I). In the subgroup with lymph node infiltration, there was no significant difference in OS between the GRID2IP-high and GRID2IP-low subgroups (may be due to insufficient sample size). Similarly, The DSS and PFI prognostic value of GRID2IP in various CRC subgroups are shown in Additional file 9: Table S8 and Additional file 10: Table S9.

**Table 4:** Prognostic value of GRID2IP (Overall Survival) in various CRC subgroups

Characteristics	*N* (%)	HR(95% CI)	*P* value
T stage			
T1&T2	131(20.4)	0.92(0.28–3.05)	0.896
T3	436 (68)	1.63(1.06–2.49)	0.025
T4	74(11.5)	1.83(0.84–3.97)	0.127
N stage			
N0	368 (57.5)	0.9(0.50–1.60)	0.716
N1	153 (23.9)	2.50(1.18–5.30)	0.017
N2	119 (18.6)	0.84(0.47–1.49)	0.551
M stage			
M0	475(84.2)	1.13(0.71–1.80)	0.614
M1	89 (15.8)	1.10(0.58–2.06)	0.776
Pathologic stage			
Stage I	111 (17.8)	0.66(0.15–2.91)	0.585
Stage II	238 (38.2)	0.78(0.39–1.56)	0.479
Stage III & Stage IV	274 (43.9)	1.84(1.16–2.91)	0.010

### GRID2IP correlated with somatic mutations and tumor burden

Microsatellite instability (MSI) is a highly mutated phenotype caused by DNA loss of mismatch repair activity, accounting for about 15% of CRCs [27]. Early CRC patients with an MSI phenotype are insensitive to chemotherapy but sensitive to immunotherapy, which generally predicts a favorable prognosis [28]. Higher tumor mutation burden (TMB) in MSI tumors is conducive to the infiltration of immunrevents tumor immune tolerance and escape, suggesting a good prognosis of CRC patients [29, 30].

**Fig. 8:**
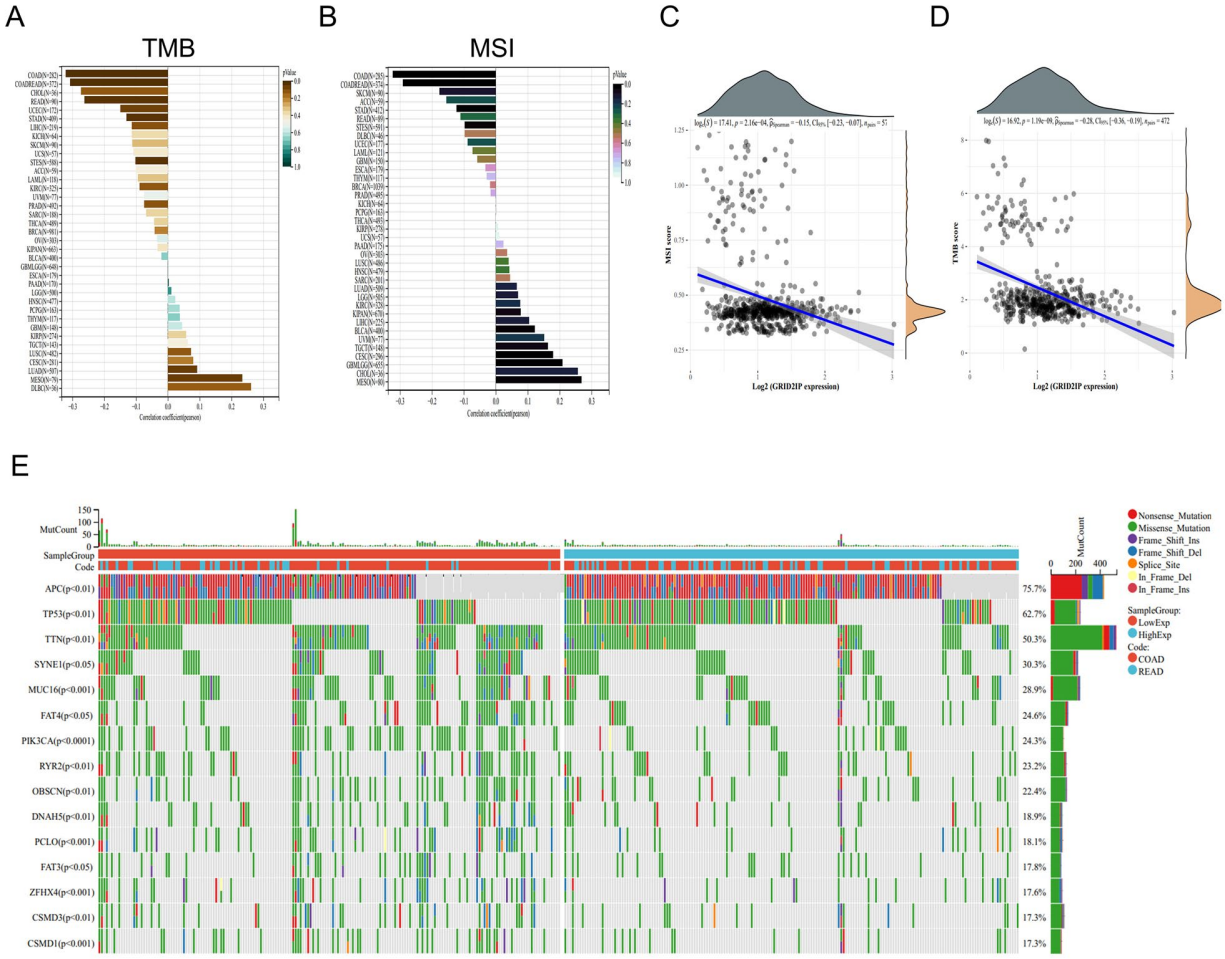
GRID2IP correlated with somatic mutations and tumor burden. **A**, **B** Bar graphs were used to visualize the pan-cancer analysis of TMB and MSI. **C**, **D** Scatter plots of correlation between TMB score, MSI score and GRID2IP expression. **E** Waterfall plots were generated to compare the differences of somatic gene mutations between different GRID2IP expression subgroups

To clarify the relationship between GRID2IP and pan-cancer TMB and MSI, we retrieved all standardized pan-cancer data sets and level4 simple nucleotide variant data sets processed by MuTect2 software from the UCSC and TCGA databases, respectively. The “tmb” function of the “maftools” package was employed to calculate the TMB of each tumor, and the TMB correlation bar graph was generated. Interestingly, we discovered a negative correlationGRID2IP expression and TMB score in CRC patients (*R* = − 0.31, *p* < 0.001) (Fig. 8A). In addition, MSI scores of tumors were extracted from previous report [31] and MSI-associated bar graph was generated (Fig. 8B). Similarly, the result show GRID2IP was significantly negatively correlated with MSI score (*R* = − 0.29, *p* < 0.001) which indicates that higher GRID2IP expression levels are associated with lower TMB and MSI scores and predicts unfavorable prognosis. Furthermore, we used Spearman to calculate the correlation of GRID2IP with TMB and MSI separately. Scatter plot results indicated that GRID2IP was significantly negatively correlated with both TMB and MSI scores (Fig. 8C, D).

To explore the somatic mutations in different GRID2IP subgroups with different expression levels, we downloaded the somatic mutation data in "raf" format from the TCGA database and generated a waterfall plot to visualize the top 15 significantly mutated genes in the high GRID2IP group and the low GRID2IP group (Fig. 8E). The results indicated that the mutation rates of *APC*, *TP53* and *TTN* were the highest, accounting for 75.7%, 62.7% and 50.3% of somatic mutations, respectively. In addition, the mutation rate of somatic cells in GRID2IP-low subgroup is higher than that in GRID2IP-high subgroup.

### Construction of prognostic prediction model

**Fig. 9:**
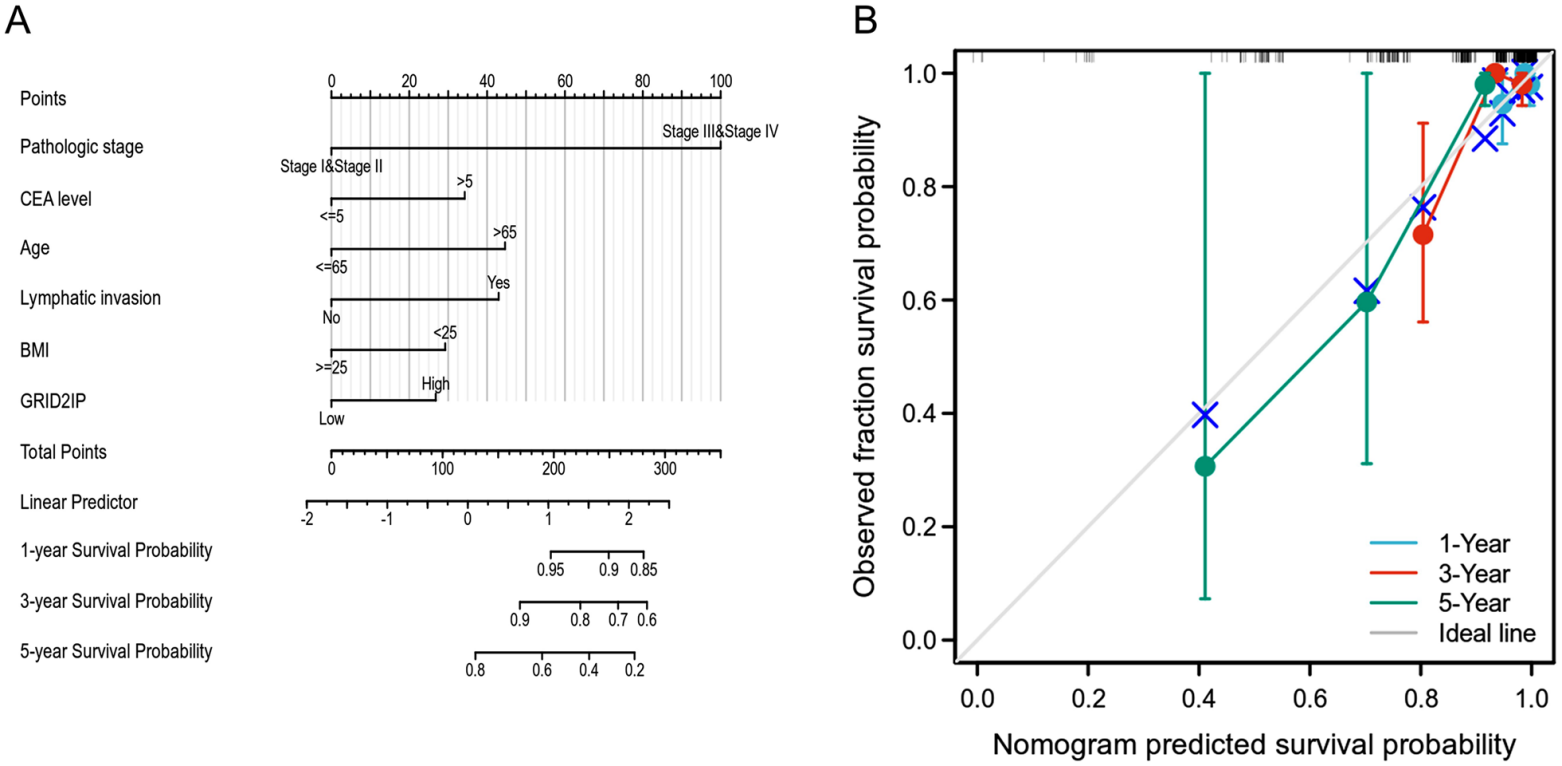
Quantitative prediction of 1-, 3-, and 5-year OS probabilities in CRC based on various clinical factors. **A** Nomogram utilized to predict 1-, 3-, and 5-year OS probability in CRC patients. **B** Calibration plot of the nomogram for predicting the OS possibility

In search of a model to predict the prognosis of CRC, we developed a nomogram based on multivariate Cox regression method. Each factor was given a quantitative score from 0 to 100, and the total score of each factor was accumulated according to its score, corresponding to the 1-, 3-, and 5-year survival probability prediction below. For example, when the age was less than 65 (45 points), pathological stage II (0 points), CEA level > 5(34 points), BMI < 25 (29 points), GRID2IP-high (37 points), and lymphatic invasion (43 points), the total score was 188 points. The corresponding 1-, 3- and 5-year survival rates were approximately 96%, 86% and 57%, respectively (Fig. 9A). In addition, we also evaluated the efficiency of the nomogram prediction model. A multivariate Cox regression analysis revealed that this prediction model's C-index was 0.796, indicating that it had a high level of accuracy, compared with the N-stage of univariate regression analysis, the results of pathologic stage, CEA level and residual tumor are more reliable (Fig. 9B). The result suggest that the nomogram prediction model is more accurate than a single factor at predicting the short- or long-term survival rate and the bad prognosis of CRC.

### Prediction of drug sensitivity

To further explore the sensitivity of patients in different GRID2IP subgroups to chemotherapy drugs, "pRRophetic" package was applied to predict the half maximal inhibitory concentration(IC50) of individual chemotherapeutic agents based on the gene expression matrix of the TCGA–COAD cohort. The results showed that compared with the GRID2IP-low subgroup, the GRID2IP-high subgroup had higher IC50 values of Gemcitabine and Cisplatin (Additional file 1: Fig. S4). This indicated that the GRID2IP-high subgroup was less sensitive to Gemcitabine and Cisplatin than the GRID2IP-low subgroup. Of note, the above results also indirectly suggest that the GRID2IP-high subgroup may have a worse prognosis.

## Discussion

As a Purkinje fiber postsynaptic scaffold protein, GRID2IP is closely related to glutamate receptor delta 2 (GRID2). Base on previous studies, GRID2 may be a biological marker for the prognosis of gastric cancer, and upregulated GRID2 is an independent risk factor for gastric cancer [32]. Therefore, we focused on the relationship of GRID2IP in CRC prognosis. High GRID2IP levels were found to be strongly associated with CRC prognosis in our study. In addition, we concentrated on the expression profile, clinicopathological significance, and clinical prognostic value of GRID2IP in CRC patients by analyzing TCGA cohort. As the result in our study, GRID2IP was substantially overexpressed in a variety of malignancies, including CRC, BRCA, CHOL, and LUAD. The ROC curve confirmed that using the expression level of GRID2IP to distinguish between normal tissue and tumor tissue has a certain specificity (AUC = 0.728). According to the GRID2IP expression level, we divided all samples into high and low GRID2IP subtypes and analyzed the DEGs between the two groups. Then, we enriched signaling pathways associated with MF, BP, CC using GO and KEGG. GSEA analysis showed that DEGs with high expression of GRID2IP were significantly enriched in GABA receptor, calcium ion and striated muscle contraction signaling pathway.

In previous studies, the occurrence and metastasis of cancer are inseparable from the immune system. Since the discovery of a major breakthrough in the use of immunotherapy to prevent and treat cancer in 1891, immunotherapy has gradually become a new cancer treatment method, such as surgery, radiotherapy, chemotherapy, and targeted therapy [33]. Each of these immune cells has its own unique function and is interconnected. It was found that DC cells, as antigen-presenting cells, can further initiate tumor-related immune responses by activating CD8 + T cells [34]. Macrophages, as indispensable immune cells in innate immunity, play a crucial role in the development and metastasis of cancer. Different types of macrophages function differently. Tumor cells are phagocytosed by pro-inflammatory M1 macrophages, whereas anti-inflammatory M2 macrophages promote tumor growth and invasion [35]. Neutrophils are not only innate immune phagocytes that act an indispensable role in immune defense but also the link between inflammation and cancer, which plays an indispensable role in the development and spread of cancer [36, 37]. In addition, Th1 cells and Th2 cells can exert potent anticancer effects by encouraging tumor interstitial remodeling and tumor tissue repair [38].

Long-term survival in CRC patients has been linked to T-cell infiltration into the tumor bed, indicating a potential function for immune regulation in regulating tumor growth [39–41]. Single-cell analysis suggested that GRID2IP was mainly expressed in immune cells, myofibroblasts and malignant cells. It is worth mentioning that the infiltration level of tumor-associated immunity, including T cells, neutrophils, macrophages, DC cells, Th1 cells, Th2 cells, T helper cells, and CD8 + T cells are significantly reduced in the GRID2IP-high group which points out that the high GRID2IP is mainly involved in the regulation of T-cell-dominated cellular immunity and macrophage-dominated innate immunity. In addition, the ESTIMATE and immune scores in the GRID2IP-high subgroup were lower than those in the GRID2IP-low subgroup, indicating a lower degree of immune cell infiltration in the GRID2IP-high subgroup. In addition, ESTIMATE score and immune score were lower in the GRID2IP-high subgroup than in the GRID2IP-low subgroup, indicating a lower degree of immune cell infiltration in the GRID2IP-high subgroup. Moreover, high GRID2IP expression was negatively correlated with most immune checkpoint inhibitor-related genes and HLA-related genes.

Another worth noting is that GRID2IP is differentially expressed in different clinical subgroups. We analyzed the differences in GRID2IP expression between different subgroups by Cox regression analysis, and found that GRID2IP was significantly correlated with some clinical features, including N-stage, M-stage, pathology stage, residual tumor, lymphatic invasion, CEA level, BMI. Moreover, the AUC diagnosed by the clinical ROC curve was 0.728, indicating that GRID2IP is a convincing biomarker for judging tumor tissue. The violin plot results indicated that patients with distant metastasis, lymph node metastasis or nodal invasion, wasting, CEA level > 5, and patients with residual tumor had poor prognosis, irrespective of gender, age and treatment effect. We also used Cox regression analysis to establish a GRID2IP-related model for predicting OS survival in CRC patients by calculating the cumulative total score for each independent prognostic factor score by nomogram. The results of the correction plots show that the model is reliable in predicting the survival rate of CRC patients.

Microsatellite instability a phenomenon in which defects in mismatch repair cause hypermutations, can be used to screen for Lynch syndrome and predict response to immune checkpoint inhibitors [42]. It is a unique biological feature of CRC, which includes high mutation burden, tumor lymphocyte infiltration and increased production of mutation-associated neoantigens [43]. Based on the previous studies, immune checkpoint inhibitors are highly effective in the treatment of CRC patients with MSI subgroups [44]. The anti-programmed death protein 1 (PD1) inhibitor pembrolizumab has been approved for first-line treatment in mismatch repair defects (dMMR) and advanced CRC in MSI-H [45].

To understand the relationship between GRID2IP and MSI status in CRC patients, we evaluated the correlation between MSI and TMB scores and GRID2IP in pan-cancer, and further visualized the correlation between MSI and TMB and GRID2IP in CRC patients with scatter plots. Specifically, we found that GRID2IP was negatively correlated with MSI and TMB in CRC patients. Subsequently, we also compared differences in somatic mutations between different GRID2IP expression subgroups by generating waterfall plot. Furthermore, we also analyzed the survival rate between GRID2IP-high and GRID2IP-low subgroups by Kaplan–Meier plot. The result show that the OS, PFI, and DSS of GRID2IP-high group are worse than the GRID2IP-low group. Grouped based on clinic pathology and independent prognostic factors, we observed T-stage (T3&T4), N-stage (N1&N2), pathology stage (T3&T4), residual tumor(R1&R2), lymphatic invasion, BMI (< 25) subgroups with high expression of GRID2IP had worse prognosis. It is further suggested that GRID2IP is a biological molecule strongly associated with the prognosis of CRC patients.

Although the above results suggest that GRID2IP is a biomarker for prognostic and therapeutic targets in CRC, our study also has certain limitations. In addition, a considerable number of cases lack complete clinical information. The therapeutic effect of GRID2IP as a therapeutic target lacks relevant reports or evidence support, and the signaling pathways involved in the occurrence and progression of CRC, as well as its upstream and downstream molecules, are not well-understood. Therefore, experimental investigation is required to determine the mechanism of action of GRID2IP on the progress of CRC.

## Conclusions

We identified that GRID2IP is substantially expressed in tumor tissues and that its expression indicates a poor prognosis for CRC in our investigation. GRID2IP plays a role in the modulation of tumor-associated immune cells. Furthermore, elevated GRID2IP suggested a worse outcome in patients with various clinical features. Meanwhile, TMB and MSI scores are associated with GRID2IP. Finally, we developed a risk factor model to predict clinical outcomes of patients in various GRID2IP expression subgroups. Taken together, GRID2IP may be a biomarker for poor prognosis as well as a possible pharmacological treatment target in CRC.

### Supplementary Information

**Additional file 1: Fig. S1.** GRID2IP protein expression in CCD18, HT19 and SW480 cells. **A** Western Blot image of GRID2IP in CCD18, HT19, and SW480 cells. **B** Gray-scale calculation of GRID2IP Western Blotting in CCD18, HT19, and SW480 cells. **Fig. S2.** Complete experimental flow chart. **Fig. S3.** Correlation between GRID2IP and TME, immune checkpoint inhibitor-related genes and HLA-related genes. **A** Relationship between GRID2IP and alterations in the immune landscape. **B** Lollipop plot of correlation between GRID2IP and immune checkpoint inhibitor-related genes. **C** Lollipop plot of correlation between GRID2IP and HLA-related genes. **Fig. S4.** Different anti-cancer drug sensitivities in GRID2IP subgroups.

**Additional file 2: Table S1. **TCGA colorectal cancer patient characteristics.

**Additional file 3: Table S2.** Top 50 of GRID2IP-related differential expressed genes.

**Additional file 4: Table S3.** GO and KEGG enrichment analysis result.

**Additional file 5. Table S4.** Results of GSEA analysis.

**Additional file 6: Table S5.** 50 items of STRING Protein interaction.

**Additional file 7: Table S6.** Univariate and Multivariate Cox regression analysis of prognostic covariates (Disease Special Survival) in patients with colorectal cancer.

**Additional file 8: Table S7.** Univariate and Multivariate Cox regression analysis of prognostic covariates (Progression Free Interval) in patients with colorectal cancer.

**Additional file 9: Table S8.** The prognostic value of GRID2IP (Disease Specific Survival) in various colorectal cancer subgroups.

**Additional file 10: Table S9.** The prognostic value of GRID2IP (Progression Free Interval) in various colorectal cancer subgroups.

## Data Availability

The raw data in the article is downloaded in the TCGA database (https://portal.gdc.cancer.gov/) or included in the Supplementary Materials section of the article. For further questions, please contact the corresponding author directly.

## Abbreviations

GRID2IP: Grid2 interacting protein
CRC: Colorectal cancer
DEGs: Differentially expressed genes
KEGG: Kyoto Encyclopedia of Genes and Genomes
GO: Gene ontology
GSEA: Gene set enrichment analysis
ssGSEA: Single-sample gene set enrichment analysis
OS: Overall survival
DSS: Disease-specific survival
PFI: Progression-free interval
HNSC: Neck squamous cell carcinoma
KICH: Renal chromophobe cell carcinoma
ROC: Receiver operating characteristic
NES: Normalized enrichment score
BLCA: Bladder carcinoma
CHOL: Cholangiocarcinoma
COAD: Colon adenocarcinoma
LUAD: Lung adenocarcinoma
AUC: Area under the curve
BP: Biological processes
CC: Cellular component
MF: Molecular function
MSI: Microsatellite instability
TMB: Tumor mutation burden
IC50: Half maximal inhibitory concentration
GRID2: Glutamate receptor delta 2
PD-1: Anti-programmed death protein 1
PPI: Protein–protein interaction
