# Supplementary material for: Grid2 interacting protein is a potential biomarker related to immune infiltration in colorectal cancer
Source: Eur J Med Res. 2023 Nov 14;28:511. doi: 10.1186/s40001-023-01468-x (PMC10644545; doi:10.1186/s40001-023-01468-x)
Supplement: Supplementary file 1 — Additional file 1: Fig. S1. GRID2IP protein expression in CCD18, HT19 and SW480 cells. A Western Blot image of GRID2IP in CCD18, HT19, and SW480 cells. B Gray-scale calculation of GRID2IP Western Blotting in CCD18, HT19, and SW480 cells. Fig. S2. Complete experimental flow chart. Fig. S3. Correlation between GRID2IP and TME, immune checkpoint inhibitor-related genes and HLA-related genes. A Relationship between GRID2IP and alterations in the immune landscape. B Lollipop plot of correlation between GRID2IP and immune checkpoint inhibitor-related genes. C Lollipop plot of correlation between GRID2IP and HLA-related genes. Fig. S4. Different anti-cancer drug sensitivities in GRID2IP subgroups. [file 40001_2023_1468_MOESM1_ESM.pdf]

Supplementary Materials

Grid2 interacting protein is a potential biomarker related to immune infiltration in colorectal cancer

Jiajing Zhao[1, †], Jiazheng Quan[2, †], Weilin Chen[2, *] & Xiaojun Xie[1, *]

[1]Department of General Surgery, the First Affiliated Hospital of Shantou University Medicine College, Shantou, 515000, China
[2]Marshall Laboratory of Biomedical Engineering, Institute of Biological Therapy, Shenzhen University Medical School, Shenzhen University, Shenzhen, 518055, China
[*]Corresponding author: Prof. Weilin Chen, Tel: 86-0755-26910334. Fax:86-0755-26910334. E-mail: cwl@szu.edu.cn.
[*]Corresponding author: Prof. Xiaojun Xie, E-mail: xjxie79@163.com
[†] These authors contributed equally to this project.

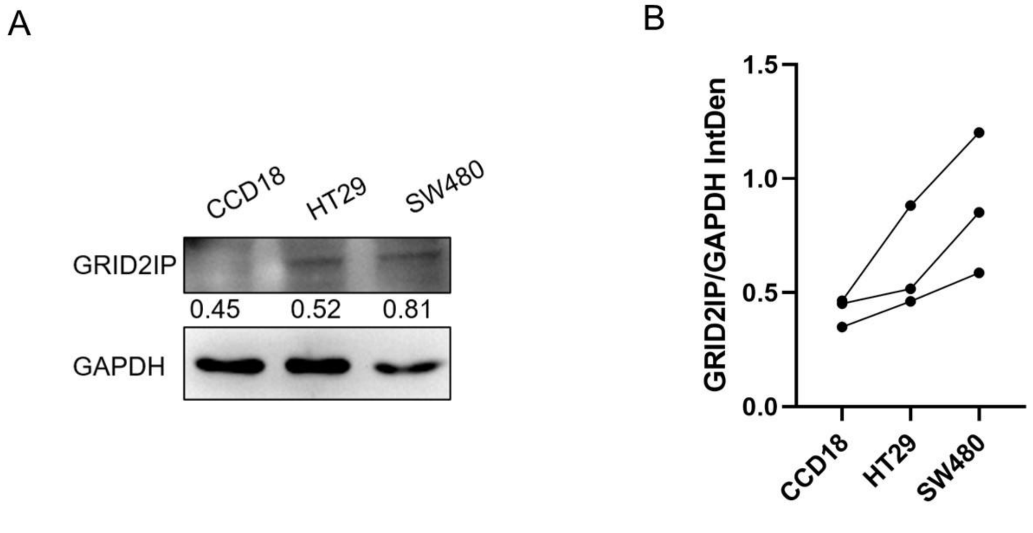

Fig. S1 GRID2IP protein expression in CCD18, HT19 and SW480 cells. **A** Western Blotting of GRID2IP in CCD18, HT19, and SW480 cells. **B** Gray-scale calculation of GRID2IP Western Blotting in CCD18, HT19, and SW480 cells

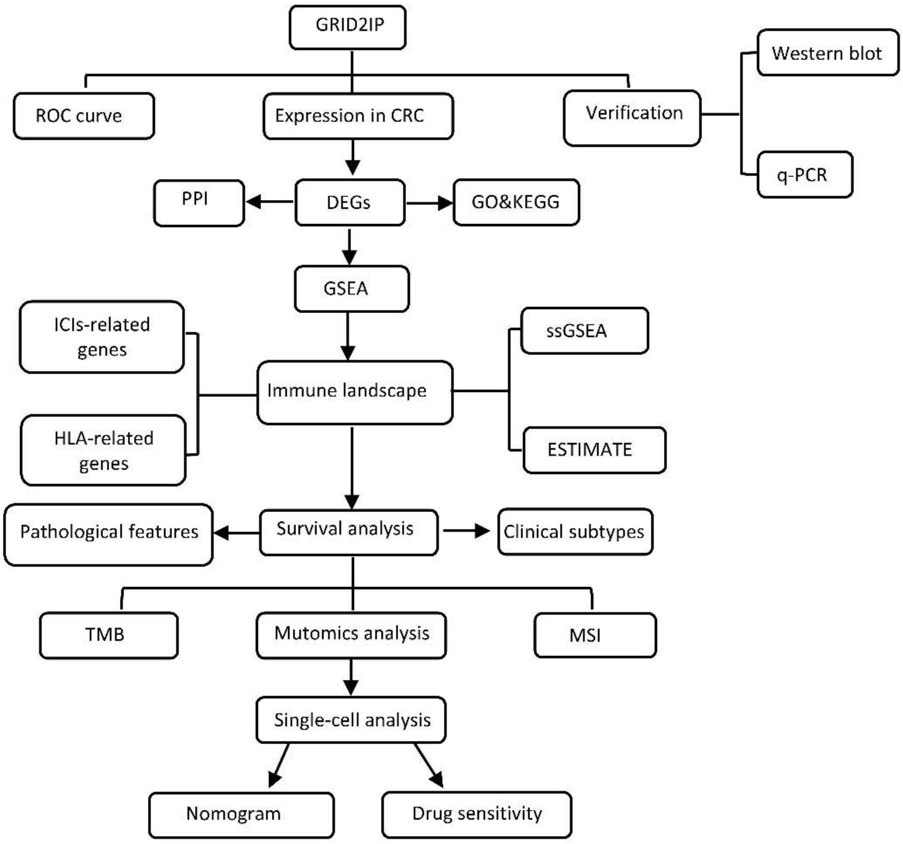

Fig. S2 Complete experimental flow chart.

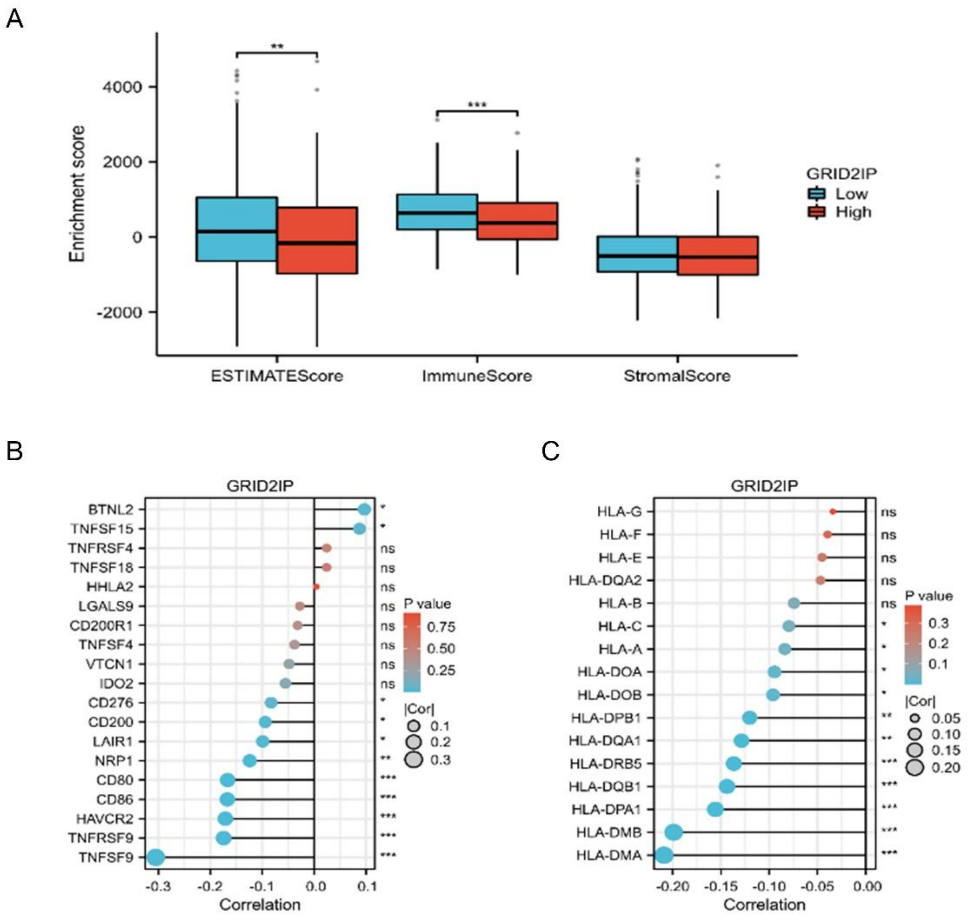

Fig. S3 Correlation between GRID2IP and TME, immune checkpoint inhibitor-related genes and HLA related genes. **A** Relationship between GRID2IP and alterations in the immune landscape. **B** Lollipop plot of correlation between GRID2IP and immune checkpoint inhibitors related genes. **C** Lollipop plot of correlation between GRID2IP and HLA related genes**.** **p*<0.05, ***p*<0.01, ****p*<0.001.

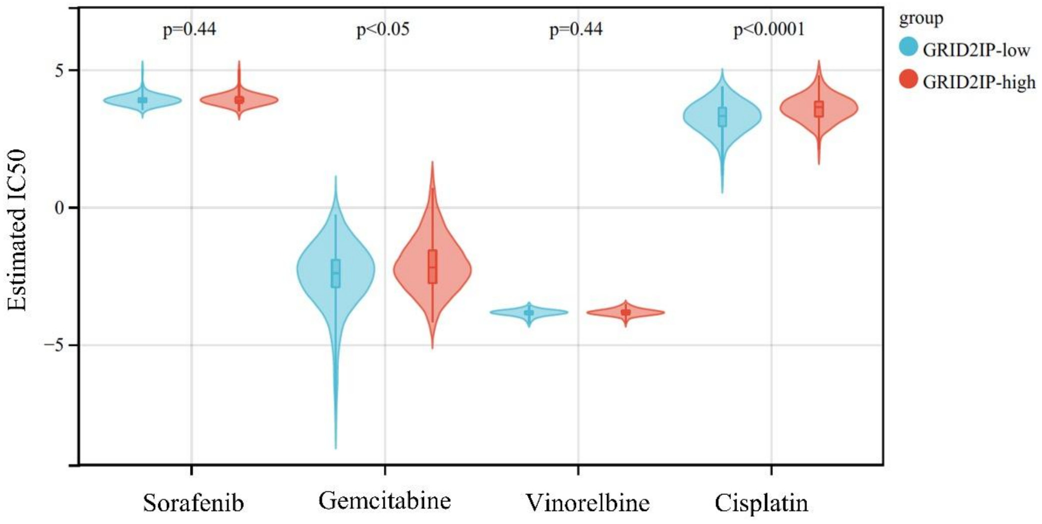

Fig. S4 Different anti-cancer drug sensitivities in GRID2IP subgroups.
