# Supplementary material for: Grid2 interacting protein is a potential biomarker related to immune infiltration in colorectal cancer
Source: Eur J Med Res. 2023 Nov 14;28:511. doi: 10.1186/s40001-023-01468-x (PMC10644545; doi:10.1186/s40001-023-01468-x)
Supplement: Supplementary file 2 — Additional file 2: Table S1. TCGA colorectal cancer patient characteristics. [file 40001_2023_1468_MOESM2_ESM.docx]

Additional file 2: Table S1: TCGA colorectal cancer patient characteristics.

| Characteristic | levels | Overall |
| --- | --- | --- |
| n |  | 644 |
| T stage, n (%) | T1 | 20 (3.1%) |
|  | T2 | 111 (17.3%) |
|  | T3 | 436 (68%) |
|  | T4 | 74 (11.5%) |
| N stage, n (%) | N0 | 368 (57.5%) |
|  | N1 | 153 (23.9%) |
|  | N2 | 119 (18.6%) |
| M stage, n (%) | M0 | 475 (84.2%) |
|  | M1 | 89 (15.8%) |
| Pathologic stage, n (%) | Stage I | 111 (17.8%) |
|  | Stage II | 238 (38.2%) |
|  | Stage III | 184 (29.5%) |
|  | Stage IV | 90 (14.4%) |
| Gender, n (%) | Female | 301 (46.7%) |
|  | Male | 343 (53.3%) |
| Race, n (%) | Asian | 12 (3%) |
|  | Black or African American | 69 (17.5%) |
|  | White | 313 (79.4%) |
| Primary therapy outcome, n (%) | PD | 33 (10.6%) |
|  | SD | 5 (1.6%) |
|  | PR | 16 (5.1%) |
|  | CR | 258 (82.7%) |
| PFI event, n (%) | Alive | 479 (74.4%) |
|  | Dead | 165 (25.6%) |
| DSS event, n (%) | Alive | 544 (87.5%) |
|  | Dead | 78 (12.5%) |
| OS event, n (%) | Alive | 515 (80%) |
|  | Dead | 129 (20%) |
| Neoplasm type, n (%) | Colon adenocarcinoma | 478 (74.2%) |
|  | Rectum adenocarcinoma | 166 (25.8%) |
| Colon polyps present, n (%) | No | 224 (69.3%) |
|  | Yes | 99 (30.7%) |
| History of colon polyps, n (%) | No | 377 (67.9%) |
|  | Yes | 178 (32.1%) |
| Lymphatic invasion, n (%) | No | 350 (60.1%) |
|  | Yes | 232 (39.9%) |
| Age, n (%) | <=65 | 276 (42.9%) |
|  | >65 | 368 (57.1%) |
| Weight, n (%) | <=90 | 244 (70.1%) |
|  | >90 | 104 (29.9%) |
| Height, n (%) | <170 | 159 (48.3%) |
|  | >=170 | 170 (51.7%) |
| BMI, n (%) | <25 | 107 (32.5%) |
|  | >=25 | 222 (67.5%) |
| Residual tumor, n (%) | R0 | 468 (91.8%) |
|  | R1 | 6 (1.2%) |
|  | R2 | 36 (7.1%) |
| CEA level, n (%) | <=5 | 261 (62.9%) |
|  | >5 | 154 (37.1%) |
| Perineural invasion, n (%) | No | 175 (74.5%) |
|  | Yes | 60 (25.5%) |
| Age, median (IQR) |  | 68 (58, 76) |
