# Supplementary material for: Grid2 interacting protein is a potential biomarker related to immune infiltration in colorectal cancer
Source: Eur J Med Res. 2023 Nov 14;28:511. doi: 10.1186/s40001-023-01468-x (PMC10644545; doi:10.1186/s40001-023-01468-x)
Supplement: Supplementary file 3 — Additional file 3: Table S2. Top 50 of GRID2IP-related differential expressed genes. [file 40001_2023_1468_MOESM3_ESM.docx]

Additional file 3: Table S2: 50 items of GRID2IP-related differential expressed Genes.

| **gene_name** | **gene_id** | **gene_biotype** | **baseMean** | **log2FoldChange** | **lfcSE** | **stat** | **pvalue** | **padj** |
| --- | --- | --- | --- | --- | --- | --- | --- | --- |
| MAGEA12 | ENSG00000213401 | protein_coding | 47.4419 | 2.827515 | 0.407661 | 6.935946 | 4.04E-12 | 2.2E-10 |
| CDH9 | ENSG00000113100 | protein_coding | 2.487664 | 2.548462 | 0.477492 | 5.337183 | 9.44E-08 | 1.58E-06 |
| CSAG1 | ENSG00000198930 | protein_coding | 21.32562 | 2.278027 | 0.345269 | 6.597835 | 4.17E-11 | 1.75E-09 |
| MAGEA9B | ENSG00000267978 | protein_coding | 1.735252 | 2.188857 | 0.580219 | 3.772466 | 0.000162 | 0.001018 |
| MAGEC2 | ENSG00000046774 | protein_coding | 2.859163 | 2.036408 | 0.493052 | 4.130207 | 3.62E-05 | 0.00028 |
| RSPO4 | ENSG00000101282 | protein_coding | 21.94553 | 2.027813 | 0.220951 | 9.177644 | 4.41E-20 | 2.11E-17 |
| MAGEA6 | ENSG00000197172 | protein_coding | 102.092 | 1.9482 | 0.418342 | 4.656958 | 3.21E-06 | 3.47E-05 |
| SFTA3 | ENSG00000229415 | protein_coding | 1.643199 | 1.926767 | 0.513655 | 3.751092 | 0.000176 | 0.001097 |
| MAGEA4 | ENSG00000147381 | protein_coding | 13.48068 | 1.914686 | 0.453117 | 4.225588 | 2.38E-05 | 0.000195 |
| MAGEB6 | ENSG00000176746 | protein_coding | 0.723288 | 1.869736 | 0.509557 | 3.669335 | 0.000243 | 0.001448 |
| GABRG3 | ENSG00000182256 | protein_coding | 1.859823 | 1.861729 | 0.297606 | 6.25568 | 3.96E-10 | 1.27E-08 |
| ZNF716 | ENSG00000182111 | protein_coding | 1.655776 | 1.859193 | 0.476012 | 3.905766 | 9.39E-05 | 0.000639 |
| MAGEA3 | ENSG00000221867 | protein_coding | 134.8796 | 1.840119 | 0.406908 | 4.522202 | 6.12E-06 | 6.08E-05 |
| SOHLH1 | ENSG00000165643 | protein_coding | 2.251456 | 1.825196 | 0.283118 | 6.446777 | 1.14E-10 | 4.27E-09 |
| LGSN | ENSG00000146166 | protein_coding | 15.9516 | 1.817954 | 0.284645 | 6.38675 | 1.69E-10 | 6.05E-09 |
| CLPSL1 | ENSG00000204140 | protein_coding | 0.88901 | 1.809923 | 0.412462 | 4.388094 | 1.14E-05 | 0.000103 |
| PAGE1 | ENSG00000068985 | protein_coding | 1.569614 | 1.803536 | 0.444461 | 4.057809 | 4.95E-05 | 0.000366 |
| AC109583.1 | ENSG00000206549 | protein_coding | 5.885883 | 1.783453 | 0.19086 | 9.344292 | 9.25E-21 | 4.86E-18 |
| ADAMTS20 | ENSG00000173157 | protein_coding | 3.091234 | 1.768446 | 0.420068 | 4.209899 | 2.55E-05 | 0.000206 |
| MAGEB1 | ENSG00000214107 | protein_coding | 0.37845 | 1.721205 | 0.969044 | 1.776188 | 0.075702 | 0.165122 |
| CLEC2L | ENSG00000236279 | protein_coding | 10.74321 | 1.682848 | 0.169455 | 9.930922 | 3.05E-23 | 2.82E-20 |
| SAGE1 | ENSG00000181433 | protein_coding | 1.736637 | 1.682204 | 0.382279 | 4.400463 | 1.08E-05 | 9.83E-05 |
| SLC10A2 | ENSG00000125255 | protein_coding | 0.95046 | 1.675228 | 0.427393 | 3.919638 | 8.87E-05 | 0.000607 |
| PRSS45P | ENSG00000188086 | protein_coding | 4.14889 | 1.660215 | 0.207536 | 7.99964 | 1.25E-15 | 1.71E-13 |
| KLK5 | ENSG00000167754 | protein_coding | 5.394677 | 1.629086 | 0.287735 | 5.661767 | 1.5E-08 | 3.11E-07 |
| NMRK2 | ENSG00000077009 | protein_coding | 1.097448 | 1.62585 | 0.287903 | 5.647214 | 1.63E-08 | 3.35E-07 |
