# Supplementary material for: Grid2 interacting protein is a potential biomarker related to immune infiltration in colorectal cancer
Source: Eur J Med Res. 2023 Nov 14;28:511. doi: 10.1186/s40001-023-01468-x (PMC10644545; doi:10.1186/s40001-023-01468-x)
Supplement: Supplementary file 4 — Additional file 4: Table S3. GO and KEGG enrichment analysis result. [file 40001_2023_1468_MOESM4_ESM.docx]

Additional file 4: Table S3：GO and KEGG enrichment analysis result

| ONTOLOGY | ID | Description | GeneRatio | BgRatio | pvalue | p.adjust | qvalue |
| --- | --- | --- | --- | --- | --- | --- | --- |
| BP | GO:0019730 | antimicrobial humoral response | 15/230 | 122/18670 | 2.77e-11 | 6.65e-08 | 6.13e-08 |
| BP | GO:0061844 | antimicrobial humoral immune response mediated by antimicrobial peptide | 12/230 | 73/18670 | 8.69e-11 | 8.24e-08 | 7.60e-08 |
| BP | GO:0070268 | cornification | 14/230 | 112/18670 | 1.03e-10 | 8.24e-08 | 7.60e-08 |
| BP | GO:0031424 | keratinization | 15/230 | 224/18670 | 1.26e-07 | 7.57e-05 | 6.98e-05 |
| BP | GO:0030216 | keratinocyte differentiation | 17/230 | 305/18670 | 2.55e-07 | 1.22e-04 | 1.13e-04 |
| CC | GO:1902711 | GABA-A receptor complex | 6/242 | 19/19717 | 7.63e-08 | 1.46e-05 | 1.19e-05 |
| CC | GO:1902710 | GABA receptor complex | 6/242 | 20/19717 | 1.08e-07 | 1.46e-05 | 1.19e-05 |
| CC | GO:0001533 | cornified envelope | 8/242 | 65/19717 | 1.27e-06 | 9.90e-05 | 8.06e-05 |
| CC | GO:0042599 | lamellar body | 5/242 | 17/19717 | 1.47e-06 | 9.90e-05 | 8.06e-05 |
| CC | GO:0032590 | dendrite membrane | 6/242 | 40/19717 | 8.70e-06 | 4.70e-04 | 3.83e-04 |
| MF | GO:0004867 | serine-type endopeptidase inhibitor activity | 11/222 | 94/17697 | 2.62e-08 | 1.04e-05 | 8.30e-06 |
| MF | GO:0004890 | GABA-A receptor activity | 6/222 | 19/17697 | 8.62e-08 | 1.72e-05 | 1.37e-05 |
| MF | GO:0016917 | GABA receptor activity | 6/222 | 22/17697 | 2.30e-07 | 3.05e-05 | 2.43e-05 |
| MF | GO:0030414 | peptidase inhibitor activity | 12/222 | 182/17697 | 3.30e-06 | 2.30e-04 | 1.83e-04 |
| MF | GO:0098960 | postsynaptic neurotransmitter receptor activity | 7/222 | 52/17697 | 3.68e-06 | 2.30e-04 | 1.83e-04 |
| KEGG | hsa04080 | Neuroactive ligand-receptor interaction | 20/98 | 341/8076 | 3.14e-09 | 4.81e-07 | 4.53e-07 |
| KEGG | hsa05033 | Nicotine addiction | 7/98 | 40/8076 | 4.19e-07 | 3.21e-05 | 3.02e-05 |
| KEGG | hsa04727 | GABAergic synapse | 8/98 | 89/8076 | 1.11e-05 | 5.66e-04 | 5.33e-04 |
| KEGG | hsa05032 | Morphine addiction | 6/98 | 91/8076 | 7.95e-04 | 0.030 | 0.029 |
| KEGG | hsa04723 | Retrograde endocannabinoid signaling | 7/98 | 148/8076 | 0.002 | 0.063 | 0.060 |
