# Supplementary material for: Grid2 interacting protein is a potential biomarker related to immune infiltration in colorectal cancer
Source: Eur J Med Res. 2023 Nov 14;28:511. doi: 10.1186/s40001-023-01468-x (PMC10644545; doi:10.1186/s40001-023-01468-x)
Supplement: Supplementary file 6 — Additional file 6: Table S5. 50 items of STRING Protein interaction. [file 40001_2023_1468_MOESM6_ESM.docx]

Additional file 6: Table S5：50 items of STRING Protein interaction.

| **node1** | **node2** | **node1_external_id** | **node2_external_id** | **neighborhood_on_chromosome** | | **gene_fusion** | **phylogenetic_cooccurrence** | **coexpression** | **experimentally_**  **determined_interaction** | **database_annotated** | **automated_textmining** | **combined_score** |
| --- | --- | --- | --- | --- | --- | --- | --- | --- | --- | --- | --- | --- |
| UPK2 | UPK1A | ENSP00000264031 | ENSP00000478942 | | 0 | 0 | 0 | 0.096 | 0.27 | 0 | 0.991 | 0.993 |
| LRP2 | ALB | ENSP00000263816 | ENSP00000295897 | | 0 | 0 | 0 | 0 | 0.213 | 0 | 0.989 | 0.991 |
| SERPINB3 | SERPINB4 | ENSP00000283752 | ENSP00000343445 | | 0 | 0 | 0.449 | 0.955 | 0.77 | 0 | 0.823 | 0.989 |
| CXCL10 | CXCL11 | ENSP00000305651 | ENSP00000306884 | | 0 | 0 | 0 | 0.841 | 0.237 | 0.9 | 0.882 | 0.988 |
| SPRR1B | SPRR1A | ENSP00000306461 | ENSP00000357751 | | 0 | 0 | 0 | 0.865 | 0 | 0.9 | 0.816 | 0.986 |
| CRP | CFHR4 | ENSP00000255030 | ENSP00000356386 | | 0 | 0 | 0 | 0.125 | 0.746 | 0 | 0.938 | 0.985 |
| SPRR2E | SPRR2A | ENSP00000357740 | ENSP00000376423 | | 0 | 0 | 0 | 0.544 | 0 | 0.9 | 0.704 | 0.985 |
| SPRR2E | SPRR2D | ENSP00000357740 | ENSP00000357746 | | 0 | 0 | 0 | 0.521 | 0 | 0.9 | 0.681 | 0.983 |
| SFTPC | SFTPB | ENSP00000316152 | ENSP00000386346 | | 0 | 0 | 0 | 0.361 | 0 | 0.6 | 0.935 | 0.982 |
| SPRR2D | SPRR2A | ENSP00000357746 | ENSP00000376423 | | 0 | 0 | 0 | 0.526 | 0 | 0.9 | 0.658 | 0.982 |
| IL1A | CXCL10 | ENSP00000263339 | ENSP00000305651 | | 0 | 0 | 0 | 0.121 | 0 | 0.9 | 0.802 | 0.981 |
| SPRR1A | SPRR2A | ENSP00000357751 | ENSP00000376423 | | 0 | 0 | 0 | 0.534 | 0 | 0.9 | 0.6 | 0.979 |
| SPRR1B | SPRR2A | ENSP00000306461 | ENSP00000376423 | | 0 | 0 | 0 | 0.534 | 0 | 0.9 | 0.564 | 0.977 |
| SPRR2G | SPRR2E | ENSP00000357737 | ENSP00000357740 | | 0 | 0 | 0 | 0.477 | 0 | 0.9 | 0.517 | 0.972 |
| SPRR1B | SPRR2D | ENSP00000306461 | ENSP00000357746 | | 0 | 0 | 0 | 0.484 | 0 | 0.9 | 0.468 | 0.97 |
| SPRR2E | SPRR1A | ENSP00000357740 | ENSP00000357751 | | 0 | 0 | 0 | 0.526 | 0 | 0.9 | 0.421 | 0.97 |
| SPRR1B | SPRR3 | ENSP00000306461 | ENSP00000330391 | | 0 | 0 | 0 | 0.679 | 0 | 0.9 | 0.816 | 0.968 |
| SPRR3 | SPRR1A | ENSP00000330391 | ENSP00000357751 | | 0 | 0 | 0 | 0.681 | 0 | 0.9 | 0.764 | 0.968 |
| SPRR3 | SPRR2D | ENSP00000330391 | ENSP00000357746 | | 0 | 0 | 0 | 0.332 | 0 | 0.9 | 0.561 | 0.968 |
| SPRR3 | SPRR2A | ENSP00000330391 | ENSP00000376423 | | 0 | 0 | 0 | 0.401 | 0 | 0.9 | 0.519 | 0.968 |
| SPRR2D | SPRR1A | ENSP00000357746 | ENSP00000357751 | | 0 | 0 | 0 | 0.414 | 0 | 0.9 | 0.504 | 0.968 |
| SPRR1B | SPRR2E | ENSP00000306461 | ENSP00000357740 | | 0 | 0 | 0 | 0.5 | 0 | 0.9 | 0.406 | 0.967 |
| SPRR3 | SPRR2E | ENSP00000330391 | ENSP00000357740 | | 0 | 0 | 0 | 0.3 | 0 | 0.9 | 0.508 | 0.962 |
| SPRR2G | SPRR2D | ENSP00000357737 | ENSP00000357746 | | 0 | 0 | 0 | 0.214 | 0 | 0.9 | 0.524 | 0.959 |
| SPRR3 | SPRR2G | ENSP00000330391 | ENSP00000357737 | | 0 | 0 | 0 | 0.102 | 0 | 0.9 | 0.509 | 0.952 |
| SPRR1B | SPRR2G | ENSP00000306461 | ENSP00000357737 | | 0 | 0 | 0 | 0.27 | 0 | 0.9 | 0.39 | 0.951 |
| KLK5 | SPINK9 | ENSP00000337733 | ENSP00000367139 | | 0 | 0 | 0 | 0 | 0 | 0.8 | 0.765 | 0.95 |
| SPRR2G | SPRR1A | ENSP00000357737 | ENSP00000357751 | | 0 | 0 | 0 | 0.345 | 0 | 0.9 | 0.282 | 0.948 |
| CACNG1 | CACNG4 | ENSP00000226021 | ENSP00000262138 | | 0 | 0.013 | 0 | 0.076 | 0 | 0.72 | 0.813 | 0.947 |
| GABRA5 | GABRA3 | ENSP00000335592 | ENSP00000359337 | | 0 | 0 | 0 | 0.13 | 0.323 | 0.9 | 0.729 | 0.937 |
| APOA4 | APOA5 | ENSP00000350425 | ENSP00000445002 | | 0 | 0 | 0 | 0.198 | 0 | 0.9 | 0.813 | 0.937 |
| REG3A | PRSS3P2 | ENSP00000377456 | ENSP00000485444 | | 0 | 0 | 0 | 0.093 | 0 | 0.9 | 0.318 | 0.932 |
| CYP1A1 | IDO1 | ENSP00000369050 | ENSP00000430950 | | 0 | 0 | 0 | 0 | 0 | 0.9 | 0.343 | 0.931 |
| MUC6 | MUC5AC | ENSP00000406861 | ENSP00000485659 | | 0 | 0 | 0 | 0.088 | 0 | 0.9 | 0.929 | 0.928 |
| SPRR2G | SPRR2A | ENSP00000357737 | ENSP00000376423 | | 0 | 0 | 0 | 0.302 | 0 | 0.9 | 0.323 | 0.927 |
| NEUROG3 | NEUROD1 | ENSP00000242462 | ENSP00000295108 | | 0 | 0 | 0 | 0 | 0 | 0.9 | 0.942 | 0.924 |
| REG1A | REG1B | ENSP00000233735 | ENSP00000303206 | | 0 | 0 | 0.447 | 0.777 | 0.66 | 0 | 0.91 | 0.922 |
| CRP | ALB | ENSP00000255030 | ENSP00000295897 | | 0 | 0 | 0 | 0.111 | 0 | 0 | 0.915 | 0.921 |
| ALDH1A2 | CYP1A1 | ENSP00000249750 | ENSP00000369050 | | 0 | 0 | 0 | 0.052 | 0.061 | 0.9 | 0.174 | 0.916 |
| LRP2 | APOA4 | ENSP00000263816 | ENSP00000350425 | | 0 | 0 | 0 | 0 | 0 | 0.9 | 0.175 | 0.914 |
| GALNTL6 | MUC5AC | ENSP00000423313 | ENSP00000485659 | | 0 | 0 | 0 | 0 | 0 | 0.9 | 0.128 | 0.909 |
| DEFA5 | PRSS3P2 | ENSP00000329890 | ENSP00000485444 | | 0 | 0 | 0 | 0 | 0.076 | 0.9 | 0.048 | 0.904 |
| GALNTL5 | MUC5AC | ENSP00000479207 | ENSP00000485659 | | 0 | 0 | 0 | 0.051 | 0 | 0.9 | 0.062 | 0.903 |
| TEX15 | HIST1H2BB | ENSP00000256246 | ENSP00000482674 | | 0 | 0 | 0 | 0.062 | 0 | 0.9 | 0 | 0.902 |
| MUC6 | GALNTL6 | ENSP00000406861 | ENSP00000423313 | | 0 | 0 | 0 | 0 | 0 | 0.9 | 0.058 | 0.901 |
| MUC6 | GALNTL5 | ENSP00000406861 | ENSP00000479207 | | 0 | 0 | 0 | 0 | 0 | 0.9 | 0 | 0.9 |
| KRT5 | KRT2 | ENSP00000252242 | ENSP00000310861 | | 0 | 0 | 0 | 0.09 | 0.837 | 0.3 | 0.599 | 0.89 |
| CSAG1 | MAGEA12 | ENSP00000359310 | ENSP00000377478 | | 0 | 0 | 0 | 0.771 | 0 | 0 | 0.518 | 0.884 |
| CSAG1 | MAGEA3 | ENSP00000359310 | ENSP00000473093 | | 0 | 0 | 0 | 0.771 | 0 | 0 | 0.509 | 0.882 |
| EPPIN | SEMG1 | ENSP00000361746 | ENSP00000361867 | | 0 | 0 | 0 | 0.062 | 0 | 0 | 0.876 | 0.879 |
