# Supplementary material for: Grid2 interacting protein is a potential biomarker related to immune infiltration in colorectal cancer
Source: Eur J Med Res. 2023 Nov 14;28:511. doi: 10.1186/s40001-023-01468-x (PMC10644545; doi:10.1186/s40001-023-01468-x)
Supplement: Supplementary file 7 — Additional file 7: Table S6. Univariate and Multivariate Cox regression analysis of prognostic covariates (Disease Special Survival) in patients with colorectal cancer. [file 40001_2023_1468_MOESM7_ESM.docx]

Additional file 7: Table S6: Univariate and Multivariate Cox regression analysis of prognostic covariates (Disease Special Survival) in patients with colorectal cancer.

| Characteristics | Total(N) | Univariate analysis | |  | Multivariate analysis | |
| --- | --- | --- | --- | --- | --- | --- |
|  |  | Hazard ratio (95% CI) | P value |  | Hazard ratio (95% CI) | P value |
| T stage | 618 |  |  |  |  |  |
| T1&T2 | 129 | Reference |  |  |  |  |
| T3&T4 | 489 | 6.440 (2.029-20.441) | 0.002 |  | 3.571 (0.462-27.620) | 0.223 |
| N stage | 617 |  |  |  |  |  |
| N0 | 358 | Reference |  |  |  |  |
| N1&N2 | 259 | 4.119 (2.496-6.797) | <0.001 |  | 0.210 (0.044-0.989) | 0.048 |
| M stage | 542 |  |  |  |  |  |
| M0 | 455 | Reference |  |  |  |  |
| M1 | 87 | 7.471 (4.647-12.012) | <0.001 |  | 2.803 (0.958-8.207) | 0.060 |
| Pathologic stage | 601 |  |  |  |  |  |
| Stage I&Stage II | 339 | Reference |  |  |  |  |
| Stage III&Stage IV | 262 | 5.716 (3.240-10.083) | <0.001 |  | 5.007 (0.697-35.961) | 0.109 |
| Residual tumor | 508 |  |  |  |  |  |
| R0 | 466 | Reference |  |  |  |  |
| R1&R2 | 42 | 6.452 (3.789-10.987) | <0.001 |  | 2.982 (1.244-7.145) | 0.014 |
| Age | 621 |  |  |  |  |  |
| <=65 | 273 | Reference |  |  |  |  |
| >65 | 348 | 1.421 (0.894-2.257) | 0.137 |  |  |  |
| BMI | 309 |  |  |  |  |  |
| <25 | 97 | Reference |  |  |  |  |
| >=25 | 212 | 1.144 (0.504-2.598) | 0.748 |  |  |  |
| CEA level | 413 |  |  |  |  |  |
| <=5 | 259 | Reference |  |  |  |  |
| >5 | 154 | 2.812 (1.566-5.050) | <0.001 |  | 1.600 (0.728-3.516) | 0.242 |
| Lymphatic invasion | 568 |  |  |  |  |  |
| No | 337 | Reference |  |  |  |  |
| Yes | 231 | 3.669 (2.241-6.006) | <0.001 |  | 2.553 (1.010-6.450) | 0.048 |
| GRID2IP | 621 |  |  |  |  |  |
| Low | 310 | Reference |  |  |  |  |
| High | 311 | 1.991 (1.248-3.175) | 0.004 |  | 1.230 (0.532-2.845) | 0.628 |

* The HR of the Reference group is the Reference, and the HR of the other groups is compared with the Reference group to obtain the corresponding HR value.
