# Supplementary material for: Grid2 interacting protein is a potential biomarker related to immune infiltration in colorectal cancer
Source: Eur J Med Res. 2023 Nov 14;28:511. doi: 10.1186/s40001-023-01468-x (PMC10644545; doi:10.1186/s40001-023-01468-x)
Supplement: Supplementary file 8 — Additional file 8: Table S7. Univariate and Multivariate Cox regression analysis of prognostic covariates (Progression Free Interval) in patients with colorectal cancer. [file 40001_2023_1468_MOESM8_ESM.docx]

Additional file 8: Table S7: Univariate and Multivariate Cox regression analysis of prognostic covariates (Progression Free Interval) in patients with colorectal cancer.

| Characteristics | Total(N) | Univariate analysis | |  | Multivariate analysis | |
| --- | --- | --- | --- | --- | --- | --- |
|  |  | Hazard ratio (95% CI) | P value |  | Hazard ratio (95% CI) | P value |
| T stage | 640 |  |  |  |  |  |
| T1&T2 | 131 | Reference |  |  |  |  |
| T3&T4 | 509 | 3.198 (1.814-5.636) | <0.001 |  | 3.624 (1.109-11.836) | 0.033 |
| N stage | 639 |  |  |  |  |  |
| N0 | 367 | Reference |  |  |  |  |
| N1&N2 | 272 | 2.624 (1.916-3.592) | <0.001 |  | 0.289 (0.065-1.284) | 0.103 |
| M stage | 563 |  |  |  |  |  |
| M0 | 474 | Reference |  |  |  |  |
| M1 | 89 | 5.577 (3.945-7.884) | <0.001 |  | 3.548 (1.609-7.827) | 0.002 |
| Pathologic stage | 622 |  |  |  |  |  |
| Stage I&Stage II | 348 | Reference |  |  |  |  |
| Stage III&Stage IV | 274 | 2.924 (2.115-4.044) | <0.001 |  | 2.186 (0.418-11.433) | 0.354 |
| Residual tumor | 509 |  |  |  |  |  |
| R0 | 467 | Reference |  |  |  |  |
| R1&R2 | 42 | 4.044 (2.605-6.280) | <0.001 |  | 1.617 (0.780-3.353) | 0.196 |
| Age | 643 |  |  |  |  |  |
| <=65 | 276 | Reference |  |  |  |  |
| >65 | 367 | 1.006 (0.737-1.371) | 0.972 |  |  |  |
| BMI | 329 |  |  |  |  |  |
| <25 | 107 | Reference |  |  |  |  |
| >=25 | 222 | 1.381 (0.857-2.224) | 0.185 |  |  |  |
| CEA level | 414 |  |  |  |  |  |
| <=5 | 260 | Reference |  |  |  |  |
| >5 | 154 | 2.628 (1.777-3.886) | <0.001 |  | 1.931 (1.160-3.215) | 0.011 |
| Lymphatic invasion | 581 |  |  |  |  |  |
| No | 349 | Reference |  |  |  |  |
| Yes | 232 | 2.358 (1.705-3.260) | <0.001 |  | 1.656 (0.946-2.899) | 0.078 |
| GRID2IP | 643 |  |  |  |  |  |
| Low | 321 | Reference |  |  |  |  |
| High | 322 | 1.398 (1.028-1.902) | 0.033 |  | 1.272 (0.759-2.132) | 0.362 |

* The HR of the Reference group is the Reference, and the HR of the other groups is compared with the Reference group to obtain the corresponding HR value.
