# Supplementary material for: Grid2 interacting protein is a potential biomarker related to immune infiltration in colorectal cancer
Source: Eur J Med Res. 2023 Nov 14;28:511. doi: 10.1186/s40001-023-01468-x (PMC10644545; doi:10.1186/s40001-023-01468-x)
Supplement: Supplementary file 9 — Additional file 9: Table S8. The prognostic value of GRID2IP (Disease Specific Survival) in various colorectal cancer subgroups. [file 40001_2023_1468_MOESM9_ESM.docx]

| Characteristics | N (%) | HR(95% CI) | P value |
| --- | --- | --- | --- |
| T stage |  |  |  |
| T1&T2 | 131(20.4) | 0.37(0.03-4.2) | 0.406 |
| T3 | 436 (68) | 2.15(1.21-3.85) | 0.010 |
| T4 | 74(11.5) | 1.92(0.83-4.45) | 0.129 |
| N stage |  |  |  |
| N0 | 368 (57.5) | 1.42(0.60-3.37) | 0.428 |
| N1 | 153 (23.9) | 3.11(1.21-8.0) | 0.018 |
| N2 | 119 (18.6) | 0.80(0.41-1.57) | 0.516 |
| M stage |  |  |  |
| M0 | 475(84.2) | 1.18(0.60-2.33) | 0.64 |
| M1 | 89 (15.8) | 1.15(0.60-2.21) | 0.666 |
| Pathologic stage |  |  |  |
| Stage I& Stage II | 349(56.0) | 0.71(0.25-2.00) | 0.517 |
| Stage III&Stage IV | 274 (43.9) | 1.97(1.15-3.39) | 0.013 |

Additional file 9: Table S8：The prognostic value of GRID2IP (Disease Specific Survival) in various colorectal cancer subgroups.
