# Supplementary material for: Grid2 interacting protein is a potential biomarker related to immune infiltration in colorectal cancer
Source: Eur J Med Res. 2023 Nov 14;28:511. doi: 10.1186/s40001-023-01468-x (PMC10644545; doi:10.1186/s40001-023-01468-x)
Supplement: Supplementary file 10 — Additional file 10: Table S9. The prognostic value of GRID2IP (Progression Free Interval) in various colorectal cancer subgroups. [file 40001_2023_1468_MOESM10_ESM.docx]

Additional file 10: Table S9：The prognostic value of GRID2IP (Progression Free Interval) in various colorectal cancer subgroups.

| Characteristics | N (%) | HR(95% CI) | P value |
| --- | --- | --- | --- |
| T stage |  |  |  |
| T1&T2 | 131(20.4) | 0.46(0.15-1.41) | 0.174 |
| T3 | 436 (68) | 1.49(1.04-2.15) | 0.031 |
| T4 | 74(11.5) | 1.47(0.73-2.95) | 0.279 |
| N stage |  |  |  |
| N0 | 368 (57.5) | 1.16(0.71-1.88) | 0.556 |
| N1 | 153 (23.9) | 1.37(0.73-2.56) | 0.332 |
| N2 | 119 (18.6) | 0.99(0.59-1.65) | 0.963 |
| M stage |  |  |  |
| M0 | 475(84.2) | 0.91(0.60-1.36) | 0.643 |
| M1 | 89 (15.8) | 1.42(0.82-2.44) | 0.209 |
| Pathologic stage |  |  |  |
| Stage I& Stage II | 349(56.0) | 1.02(0.61-1.71) | 0.936 |
| Stage III&Stage IV | 274 (43.9) | 1.48(1.00-2.00) | 0.048 |
