# Supplementary material for: Grid2 interacting protein is a potential biomarker related to immune infiltration in colorectal cancer
Source: Eur J Med Res. 2023 Nov 14;28:511. doi: 10.1186/s40001-023-01468-x (PMC10644545; doi:10.1186/s40001-023-01468-x)
Supplement: Supplementary file 10 [file 40001_2023_1468_MOESM5_ESM.docx]

ID	setSize	enrichmentScore	NES	pvalue	p.adjust	qvalues	rank	leading_edge	core_enrichment
REACTOME_PHASE_0_RAPID_DEPOLARISATION	42	0.6832661497977	2.08	0.0015	0.06127803	0.0481529468	3268	tags=48%, list=17%, signal=40%	CACNG4/SCN5A/CAMK2B/CACNA1F/SCN3B/FGF13/SCN4A/SCN1A/SCN7A/CACNG2/CACNA1S/CACNA1D/CACNA2D4/SCN11A/SCN2A/FGF14/SCN8A/CACNG3/SCN2B/SCN4B
WP_PRADERWILLI_AND_ANGELMAN_SYNDROME	61	0.622671380592696	2.04	0.0014	0.06127803	0.0481529468	2176	tags=36%, list=11%, signal=32%	GABRG3/GABRG1/GABRR1/GABRA5/GABRG2/HTR2C/NPAP1/KISS1/INS/TACR3/OCA2/DLX5/CDKN2A/MKRN3/SLC45A2/NHLH2/GABRD/SNURF/GHRH/SNRPN/OXT/NGF
WP_GABA_RECEPTOR_SIGNALING	31	0.689311035150515	1.99	0.0015	0.06127803	0.0481529468	1953	tags=42%, list=10%, signal=38%	GABRG3/GABRG1/GABRA3/GABRA5/GAD2/GABRG2/GABRQ/ABAT/GABRE/GABRA1/GABRA6/GABRD/GABRA2
KEGG_NEUROACTIVE_LIGAND_RECEPTOR_INTERACTION	270	0.486583555251694	1.91	0.0011	0.06127803	0.0481529468	2345	tags=34%, list=12%, signal=31%	GABRG3/GABRG1/HRH3/GABRA3/GABRR1/GABRA5/GRPR/P2RX3/PRLHR/CHRNA4/NPY5R/MCHR2/DRD2/CCKBR/GABRG2/MC3R/HTR2C/CRHR2/CHRNA2/LEP/DRD1/NTSR1/AVPR2/GLP1R/GLRA2/GRIN2C/NPY1R/GRIA2/NPFFR1/OPRM1/AGTR2/DRD5/CALCR/CRHR1/GPR156/THRB/GRIN2A/MC1R/GABRQ/GLRA3/CHRNG/MC4R/SCTR/GABRE/CNR1/LHCGR/GRIN3B/VIPR2/TACR2/GHRHR/TACR3/TRPV1/MAS1/PTH1R/GLRA1/GABRA1/GRIA1/AGTR1/SSTR5/UTS2R/NMUR1/CHRNB3/HTR1E/P2RX2/GRM8/DRD4/GRIK5/GRM3/PLG/GPR83/CHRNB2/MC2R/VIPR1/OPRL1/ADCYAP1R1/LTB4R/GABRA6/GABRD/TSHB/F2/BRS3/GRIK4/GABRA2/NPFFR2/GRM7/HCRTR2/DRD3/ADORA2A/HTR5A/CHRM2/ADRA1A/P2RX5/PTGER1
REACTOME_VOLTAGE_GATED_POTASSIUM_CHANNELS	42	0.625739651031721	1.9	0.0015	0.06127803	0.0481529468	1767	tags=40%, list=9%, signal=37%	KCNH5/KCNH6/KCNG1/KCNG4/KCNA4/KCNH2/KCNB1/KCNA2/KCNF1/KCNH4/KCNS2/KCNC2/KCNH3/KCNH8/KCNC1/KCNQ2/KCNAB2
REACTOME_LGI_ADAM_INTERACTIONS	14	0.78559442672039	1.89	0.0033	0.06127803	0.0481529468	1878	tags=57%, list=10%, signal=52%	CACNG4/LGI3/LGI4/LGI1/STX1A/ADAM11/CACNG2/STX1B
WP_STRIATED_MUSCLE_CONTRACTION_PATHWAY	38	0.615084642297584	1.83	0.003	0.06127803	0.0481529468	4144	tags=55%, list=22%, signal=43%	MYH6/TNNC2/ACTN2/DES/TNNC1/MYL3/MYBPC1/TNNT2/MYH3/TNNT3/DMD/ACTA1/NEB/MYOM1/TCAP/MYBPC2/TNNI3/MYH8/TPM2/TNNI1/MYL9
REACTOME_GAMMA_CARBOXYLATION_TRANSPORT_AND_AMINO_TERMINAL_CLEAVAGE_OF_PROTEINS	11	0.8052186852113	1.83	0.0066	0.0639946	0.0502876625	1852	tags=55%, list=10%, signal=49%	F7/PROC/F10/PROZ/BGLAP/F2
REACTOME_STRIATED_MUSCLE_CONTRACTION	36	0.614661730790895	1.82	0.0045	0.06127803	0.0481529468	4058	tags=56%, list=21%, signal=44%	MYH6/TNNC2/ACTN2/DES/TNNC1/MYL3/MYBPC1/TNNT2/MYH3/TNNT3/DMD/ACTA1/NEB/TCAP/MYBPC2/TNNI3/MYH8/TMOD2/TPM2/TNNI1
REACTOME_REMOVAL_OF_AMINOTERMINAL_PROPEPTIDES_FROM_GAMMA_CARBOXYLATED_PROTEINS	10	0.813974347942066	1.82	0.0066	0.0639946	0.0502876625	1852	tags=60%, list=10%, signal=54%	F7/PROC/F10/PROZ/BGLAP/F2

REACTOME_VOLTAGE_GATED_POTASSIUM_CHANNELS	42	0.625739651031721	1.9	0.0015	0.06127803	0.0481529468	1767	tags=40%, list=9%, signal=37%	KCNH5/KCNH6/KCNG1/KCNG4/KCNA4/KCNH2/KCNB1/KCNA2/KCNF1/KCNH4/KCNS2/KCNC2/KCNH3/KCNH8/KCNC1/KCNQ2/KCNAB2
REACTOME_LGI_ADAM_INTERACTIONS	14	0.78559442672039	1.89	0.0033	0.06127803	0.0481529468	1878	tags=57%, list=10%, signal=52%	CACNG4/LGI3/LGI4/LGI1/STX1A/ADAM11/CACNG2/STX1B
WP_STRIATED_MUSCLE_CONTRACTION_PATHWAY	38	0.615084642297584	1.83	0.003	0.06127803	0.0481529468	4144	tags=55%, list=22%, signal=43%	MYH6/TNNC2/ACTN2/DES/TNNC1/MYL3/MYBPC1/TNNT2/MYH3/TNNT3/DMD/ACTA1/NEB/MYOM1/TCAP/MYBPC2/TNNI3/MYH8/TPM2/TNNI1/MYL9
REACTOME_GAMMA_CARBOXYLATION_TRANSPORT_AND_AMINO_TERMINAL_CLEAVAGE_OF_PROTEINS	11	0.8052186852113	1.83	0.0066	0.0639946	0.0502876625	1852	tags=55%, list=10%, signal=49%	F7/PROC/F10/PROZ/BGLAP/F2
REACTOME_STRIATED_MUSCLE_CONTRACTION	36	0.614661730790895	1.82	0.0045	0.06127803	0.0481529468	4058	tags=56%, list=21%, signal=44%	MYH6/TNNC2/ACTN2/DES/TNNC1/MYL3/MYBPC1/TNNT2/MYH3/TNNT3/DMD/ACTA1/NEB/TCAP/MYBPC2/TNNI3/MYH8/TMOD2/TPM2/TNNI1
REACTOME_REMOVAL_OF_AMINOTERMINAL_PROPEPTIDES_FROM_GAMMA_CARBOXYLATED_PROTEINS	10	0.813974347942066	1.82	0.0066	0.0639946	0.0502876625	1852	tags=60%, list=10%, signal=54%	F7/PROC/F10/PROZ/BGLAP/F2
WP_MBDNF_AND_PROBDNF_REGULATION_OF_GABA_NEUROTRANSMISSION	38	0.601753902473809	1.79	0.003	0.06127803	0.0481529468	1953	tags=34%, list=10%, signal=31%	GABRG3/GABRG1/GABRA3/GABRA5/GABRG2/GABRQ/GABRE/PLCG1/GABRA1/GABRA6/GABRD/NGFR/GABRA2
REACTOME_DISEASES_ASSOCIATED_WITH_SURFACTANT_METABOLISM	10	0.798820937996033	1.78	0.0066	0.0639946	0.0502876625	2007	tags=60%, list=11%, signal=54%	SFTA3/SFTPC/SFTPB/SFTPA1/SLC34A2/SFTPD
REACTOME_INTERACTION_BETWEEN_L1_AND_ANKYRINS	31	0.615306404541959	1.77	0.0061	0.06144716	0.0482858522	3367	tags=52%, list=18%, signal=43%	SCN5A/L1CAM/SCN3B/SCN4A/SCN1A/SPTBN4/SCN7A/NFASC/SCN11A/KCNQ2/SPTBN5/SCN2A/SCN8A/SCN2B/SCN4B/SPTBN2
WP_GPCRS_CLASS_B_SECRETINLIKE	24	0.643041725289979	1.75	0.0062	0.06155244	0.048368585	1706	tags=46%, list=9%, signal=42%	CRHR2/GLP1R/CALCR/CRHR1/SCTR/VIPR2/GHRHR/ADGRG2/PTH1R/VIPR1/ADCYAP1R1
REACTOME_SURFACTANT_METABOLISM	30	0.596882084148171	1.71	0.0106	0.07181584	0.0564336785	2135	tags=30%, list=11%, signal=27%	SFTA3/SFTPC/SFTPB/SFTPA1/NAPSA/SLC34A1/SLC34A2/SFTPD/ADORA2A
REACTOME_REGULATION_OF_SIGNALING_BY_NODAL	11	0.742265776232125	1.69	0.0115	0.07553223	0.0593540611	3139	tags=64%, list=16%, signal=53%	DAND5/NODAL/LEFTY2/LEFTY1/ACVR2B/ACVR1B/TDGF1
REACTOME_CLASS_B_2_SECRETIN_FAMILY_RECEPTORS_	94	0.482332864981516	1.69	0.0026	0.06127803	0.0481529468	2369	tags=33%, list=12%, signal=29%	CRHR2/GNG4/WNT7A/GLP1R/CALCR/GNGT1/CRHR1/FZD10/IHH/CRH/SCTR/PTCH2/VIPR2/GHRHR/FZD7/PTH1R/VIP/SCT/WNT16/VIPR1/UCN/ADCYAP1R1/SMO/WNT11/GHRH/SHH/PTCH1/WNT1/IAPP/WNT10A/WNT8A
REACTOME_CARDIAC_CONDUCTION	137	0.457813849311494	1.68	0.0013	0.06127803	0.0481529468	4843	tags=46%, list=25%, signal=35%	CACNG4/SCN5A/SLC8A2/CAMK2B/CASQ2/KCNK17/CACNA1F/KCNH2/SCN3B/ATP1A2/FGF13/SCN4A/KCNK16/SCN1A/KCNK9/RYR1/ATP1A4/SCN7A/KCNK5/CASQ1/KCNIP3/CACNG2/CACNA1S/CACNA1D/CACNA2D4/SCN11A/KCNIP2/KCNJ12/KCNK15/ATP2B2/SCN2A/FXYD2/FGF14/TRPC1/NOS1/ATP1A3/FXYD1/ORAI2/FXYD7/SCN8A/KCNJ14/CACNG3/KCNK3/SCN2B/KCNIP1/SCN4B/NPPC/TNNI3/SRI/KCNK12/RYR2/FKBP1B/AKAP9/DMPK/ATP2B3/CACNB1/KCNQ1/KCNJ11/FXYD6/ATP2A1/KCND1/ATP1B2/TRDN
REACTOME_MUSCLE_CONTRACTION	205	0.440532739183198	1.68	0.0012	0.06127803	0.0481529468	4285	tags=40%, list=23%, signal=32%	CACNG4/SCN5A/MYH6/SLC8A2/CAMK2B/TNNC2/CASQ2/ACTN2/KCNK17/DES/TNNC1/CACNA1F/KCNH2/MYL3/SCN3B/MYH11/ATP1A2/FGF13/SCN4A/MYBPC1/KCNK16/TNNT2/MYH3/SCN1A/KCNK9/ACTG2/TNNT3/RYR1/ATP1A4/SCN7A/KCNK5/DMD/CASQ1/KCNIP3/CACNG2/CACNA1S/CACNA1D/CACNA2D4/ACTA1/SCN11A/KCNIP2/KCNJ12/SORBS1/KCNK15/ATP2B2/SCN2A/FXYD2/FGF14/TRPC1/NOS1/ATP1A3/FXYD1/NEB/ORAI2/MYL7/FXYD7/LMOD1/MYLPF/SCN8A/KCNJ14/CACNG3/KCNK3/SCN2B/KCNIP1/TCAP/SCN4B/MYBPC2/MYL10/NPPC/TNNI3/MYH8/SRI/TMOD2/KCNK12/TPM2/ITGB5/TNNI1/MYL9/RYR2/FKBP1B/AKAP9/DMPK/ATP2B3
WP_RETT_SYNDROME_CAUSING_GENES	48	0.537557983211954	1.68	0.0088	0.06635261	0.0521406127	1940	tags=23%, list=10%, signal=21%	GABRA3/CECR2/FOXG1/GRIN2A/SCN1A/HDAC5/HAP1/ACTL6B/GABRD/SRRM3/SCN2A

REACTOME_NEURONAL_SYSTEM	409	0.414625755814664	1.68	0.0011	0.06127803	0.0481529468	2991	tags=28%, list=16%, signal=24%	GABRG3/GABRA3/CACNG4/KCNH5/GABRR1/GABRA5/TUBA3C/CHRNA4/GAD2/GABRG2/SLC6A4/CAMK2B/SYN3/NRXN2/CHRNA2/GNG4/ACTN2/RPS6KA6/KCNH6/KCNG1/KCNK17/KCNG4/GLRA2/ADCY8/GRIN2C/GRIA2/KCNA4/KCNH2/KCNB1/ERBB4/KCNK16/LRRTM1/SLC18A3/GNGT1/KCNA2/GRIN2A/KCNF1/KCNH4/STX1A/KCNS2/SLC5A7/KCNC2/PRKAG3/GABRQ/GLRA3/CHRNG/SLC1A7/NTRK3/KCNK9/ABAT/PPM1E/DLGAP2/TUBA3E/EPB41L1/KCNH3/GRIN3B/ACHE/SYT7/KCNMB3/RIMS1/KCNH8/KCNC1/LRFN2/GLRA1/SLITRK1/GABRA1/GRIA1/GJD2/SHANK2/CHRNB3/LRRTM4/CACNG2/GRIK5/ADCY5/SNAP25/SYT2/CHRNB2/LIN7B/HCN2/KCNJ12/GABRA6/KCNQ2/KCNAB2/HCN3/SLC6A3/TUBB8B/LRRC4B/GRIK4/GABRA2/PRKAR1B/PPFIA3/TUBA3D/HOMER3/PPFIA4/CPLX1/DNAJC5/SRC/SLC1A6/TUBB1/GRIA4/NLGN1/GRM5/RASGRF1/NRXN1/ADCY3/LRFN1/DLGAP3/KCNJ14/PANX2/CACNG3/KCNK3/TUBA4B/DLGAP1/PLCB1/TUBB8
REACTOME_ACTIVATION_OF_THE_PHOTOTRANSDUCTION_CASCADE	11	0.735787230128614	1.67	0.0148	0.08559134	0.0672586185	3329	tags=64%, list=17%, signal=53%	GNGT1/SAG/PDE6A/PDE6B/CNGA1/CNGB1/GNAT1
WP_THE_ALTERNATIVE_PATHWAY_OF_FETAL_ANDROGEN_SYNTHESIS	11	0.733889711425447	1.67	0.0148	0.08559134	0.0672586185	2721	tags=73%, list=14%, signal=62%	CYP17A1/HSD17B3/CYP11A1/SRD5A2/AKR1C2/STAR/AKR1C4/POR
KEGG_BASAL_CELL_CARCINOMA	55	0.520474361562637	1.67	0.0087	0.06635261	0.0521406127	3516	tags=40%, list=18%, signal=33%	WNT7A/FZD10/PTCH2/FZD7/WNT5B/WNT16/SMO/WNT11/SHH/HHIP/PTCH1/WNT1/WNT10A/WNT8A/STK36/AXIN1/APC2/WNT3/TCF7L1/WNT3A/TCF7/AXIN2
BIOCARTA_GABA_PATHWAY	10	0.7382476819834	1.65	0.0231	0.11359444	0.0892637631	1953	tags=50%, list=10%, signal=45%	GABRA3/GABRA5/GABRA1/GABRA6/GABRA2
REACTOME_ACETYLCHOLINE_NEUROTRANSMITTER_RELEASE_CYCLE	17	0.653497875865886	1.64	0.0182	0.09715114	0.0763424329	2514	tags=47%, list=13%, signal=41%	SLC18A3/STX1A/SLC5A7/RIMS1/SNAP25/PPFIA3/PPFIA4/CPLX1
WP_NICOTINE_ACTIVITY_ON_DOPAMINERGIC_NEURONS	21	0.62377678243749	1.64	0.0111	0.07349952	0.0577567314	2904	tags=43%, list=15%, signal=36%	CHRNA4/DRD2/KCNK9/DRD4/CHRNB2/DDC/DRD3/TH/KCNK3
REACTOME_ADHERENS_JUNCTIONS_INTERACTIONS	33	0.564393487235849	1.64	0.0121	0.07754828	0.0609382948	1917	tags=21%, list=10%, signal=19%	CDH9/CDH12/CDH10/CDH18/CADM2/CDH8/CDH4

REACTOME_SODIUM_CALCIUM_EXCHANGERS	11	0.710999383337139	1.62	0.0231	0.11345506	0.0891542329	160	tags=18%, list=1%, signal=18%	SLC24A5/SLC8A2
KEGG_CALCIUM_SIGNALING_PATHWAY	177	0.429300991724347	1.61	0.0012	0.06127803	0.0481529468	3491	tags=31%, list=18%, signal=25%	GRPR/P2RX3/CCKBR/SLC8A2/CAMK2B/HTR2C/TNNC2/DRD1/NTSR1/TNNC1/ADCY8/CACNA1F/GRIN2C/DRD5/CHP2/ERBB4/CALML6/GRIN2A/LHCGR/TACR2/RYR1/PPP3R2/TACR3/PLCG1/AGTR1/P2RX2/CACNA1S/CACNA1G/PHKG1/CACNA1D/PHKA2/ATP2B2/ADORA2A/HTR5A/MYLK3/CHRM2/ADRA1A/TRPC1/P2RX5/PLCB4/PTGER1/NOS1/GRM5/CHRM5/ADCY3/EGFR/PLCB1/SLC25A31/PLCD1/ERBB3/ADCY1/SLC25A6/CCKAR/P2RX6
REACTOME_NEGATIVE_REGULATION_OF_NMDA_RECEPTOR_MEDIATED_NEURONAL_TRANSMISSION	21	0.608506821130325	1.6	0.0223	0.11040304	0.086755927	820	tags=24%, list=4%, signal=23%	CAMK2B/ACTN2/GRIN2C/GRIN2A/PPM1E
REACTOME_GABA_RECEPTOR_ACTIVATION	60	0.488057230518459	1.59	0.0143	0.08461994	0.0664952817	1953	tags=23%, list=10%, signal=21%	GABRG3/GABRA3/GABRR1/GABRA5/GABRG2/GNG4/ADCY8/GNGT1/GABRQ/GABRA1/ADCY5/KCNJ12/GABRA6/GABRA2
WP_SYNAPTIC_VESICLE_PATHWAY	51	0.502947475657551	1.59	0.0163	0.09188854	0.0722070261	2514	tags=29%, list=13%, signal=26%	CPLX2/UNC13C/SLC6A4/SYN3/ATP1A2/SLC18A3/STX1A/SYP/DNM3/RIMS1/SNAP25/STX1B/SLC17A6/SLC22A3/CPLX1
REACTOME_NEUROTRANSMITTER_RECEPTORS_AND_POSTSYNAPTIC_SIGNAL_TRANSMISSION	205	0.414058624286363	1.58	0.0012	0.06127803	0.0481529468	1964	tags=21%, list=10%, signal=19%	GABRG3/GABRA3/CACNG4/GABRR1/GABRA5/TUBA3C/CHRNA4/GABRG2/CAMK2B/CHRNA2/GNG4/ACTN2/RPS6KA6/GLRA2/ADCY8/GRIN2C/GRIA2/ERBB4/GNGT1/GRIN2A/PRKAG3/GABRQ/GLRA3/CHRNG/PPM1E/TUBA3E/EPB41L1/GRIN3B/GLRA1/GABRA1/GRIA1/CHRNB3/CACNG2/GRIK5/ADCY5/CHRNB2/LIN7B/KCNJ12/GABRA6/TUBB8B/GRIK4/GABRA2/PRKAR1B
REACTOME_NEUROTOXICITY_OF_CLOSTRIDIUM_TOXINS	10	0.708076051328236	1.58	0.0364	0.15704587	0.1234083719	1878	tags=50%, list=10%, signal=45%	SV2C/STX1A/SNAP25/SYT2/STX1B
REACTOME_INTRINSIC_PATHWAY_OF_FIBRIN_CLOT_FORMATION	22	0.588336565567105	1.57	0.0301	0.13686808	0.1075524451	3661	tags=55%, list=19%, signal=44%	PROC/F10/KNG1/GP5/F11/SERPINE2/SERPIND1/F2/F8/F9/SERPINA5/KLKB1
PID_WNT_SIGNALING_PATHWAY	27	0.560070698890035	1.57	0.0234	0.11411932	0.0896762157	3343	tags=33%, list=18%, signal=28%	WIF1/WNT7A/FZD10/FZD7/WNT1/LRP5/KREMEN2/WNT3/WNT3A
REACTOME_CELL_CELL_JUNCTION_ORGANIZATION	64	0.471585937624113	1.55	0.02	0.1038834	0.0816327158	1917	tags=23%, list=10%, signal=21%	CDH9/CDH12/CLDN9/CDH10/CDH18/CLDN19/CADM2/CLDN11/CLDN15/CLDN6/CLDN3/CLDN4/CDH8/CLDN16/CDH4
REACTOME_AMINE_LIGAND_BINDING_RECEPTORS	42	0.508940239773537	1.55	0.0196	0.10331155	0.0811833505	3604	tags=38%, list=19%, signal=31%	HRH3/DRD2/HTR2C/DRD1/DRD5/GPR143/HTR1E/DRD4/DRD3/HTR5A/CHRM2/ADRA1A/CHRM5/ADRA2B/CHRM4/ADRA1D
KEGG_DILATED_CARDIOMYOPATHY	90	0.448257504516589	1.55	0.0067	0.0639946	0.0502876625	4479	tags=37%, list=24%, signal=28%	CACNG4/CACNG1/MYH6/DES/TNNC1/ADCY8/CACNA1F/MYL3/TNNT2/DMD/CACNG2/CACNA1S/ADCY5/SGCA/CACNA1D/CACNA2D4/SGCG/ITGA10/ITGA9/ADCY3/ITGA7/CACNG3/ITGB8/ADCY1/TNNI3/EMD/TPM2/ITGB5/RYR2/GNAS/PRKACG/TGFB2/CACNB1
REACTOME_REGULATION_OF_FZD_BY_UBIQUITINATION	21	0.587384058867238	1.55	0.0302	0.13711543	0.1077468133	3343	tags=38%, list=18%, signal=31%	RSPO4/RSPO2/LGR6/RNF43/LGR5/ZNRF3/LRP5/WNT3A
